# Transactivation of *Sus1* and *Sus2* by Opaque2 is an essential supplement to sucrose synthase‐mediated endosperm filling in maize

**DOI:** 10.1111/pbi.13349

**Published:** 2020-03-26

**Authors:** Yiting Deng, Jiechen Wang, Zhiyong Zhang, Yongrui Wu

**Affiliations:** ^1^ National Key Laboratory of Plant Molecular Genetics CAS Center for Excellence in Molecular Plant Sciences Institute of Plant Physiology & Ecology Shanghai Institutes for Biological Sciences Chinese Academy of Sciences Shanghai China; ^2^ University of the Chinese Academy of Sciences Beijing China

**Keywords:** O2, Quality Protein Paize, *Sh1*, *Sus1*, *Sus2*, starch, storage protein, endosperm

## Abstract

The endosperm‐specific transcription factor Opaque2 (O2) acts as a central regulator for endosperm filling, but its functions have not been fully defined. Regular *o2* mutants exhibit a non‐vitreous phenotype, so we used its vitreous variety Quality Protein Maize to create EMS‐mutagenesis mutants for screening *o2* enhancers (*oen*). A mutant (*oen1*) restored non‐vitreousness and produced a large cavity in the seed due to severely depleted endosperm filling. When *oen1* was introgressed into inbred W64A with a normal *O2* gene, the seeds appeared vitreous but had a shrunken crown. *oen1* was determined to encode Shrunken1 (Sh1), a sucrose synthase (SUS, EC 2.4.1.13). Maize contains three SUS‐encoding genes (*Sh1*, *Sus1*, and *Sus2*) with *Sh1* contributing predominantly to the endosperm. We determined SUS activity and found a major and minor reduction in *oen1* and *o2*, respectively. In *o2;oen1-1*, SUS activity was further decreased. We found all *Sus* gene promoters contain at least one O2 binding element that can be specifically recognized and be transactivated by O2. *Sus1* and *Sus2* promoters had a much stronger O2 transactivation than *Sh1*, consistent with their transcript reduction in *o2* endosperm. Although *sus1* and *sus2* alone or in combination had no perceptible phenotype, either of them could dramatically enhance seed opacity and cavity in *sh1*, indicating that transactivation of *Sus1* and *Sus2* by O2 supplements SUS‐mediated endosperm filling in maize. Our findings demonstrate that O2 transcriptionally regulates the metabolic source entry for protein and starch synthesis during endosperm filling.

## Introduction

The nutrients transported into maize grain during filling are derived from photoassimilated products in leaves. Sucrose is the main photosynthate product for long‐distance transport to the terminal sink tissue, the developing seed (Schleucher *et al.*, 1998). When sucrose enters the seed, two enzymatic pathways are known to catalyse its cleavage, one involving cell wall invertase 2 (CWI2), which breaks down sucrose into fructose and glucose (Kang *et al.*, 2009), and the other involving sucrose synthase (SUS), which converts sucrose plus uridine diphosphate (UDP) to fructose and UDP‐glucose (Chourey and Nelson, 1979; Koch, 2004; Winter and Huber, 2000). CWI2‐mediated sucrose cleavage occurs at the basal endosperm transfer layer (BETL; Kang *et al.*, 2009). The *cwi2* mutant (known as *minature1*, *mn1*) exhibits greatly reduced endosperm size (Cheng *et al.*, 1996). Although the products of CWI2 make contributions to basic precursors for grain filling, sucrose can also directly enter the endosperm cells without hydrolysis (Cobb and Hannah, 1986; Schmalstig and Hitz, 1987). Sucrose can be converted to fructose and UDP‐glucose by the action of SUS, providing substrates for the synthesis of starch and proteins. Although the action of SUS is reversible, that is UDP‐glucose and fructose can also be resynthesized to sucrose, sucrose hydrolysis predominates over resynthesis, probably due to the lack of oxygen and ATP in developing endosperm cells (Borisjuk and Rolletschek, 2009; Rolletschek *et al.*, 2005). The conversion of sucrose to UDP‐glucose and fructose is advantageous to endosperm cells, as it reduces the consumption of oxygen and ATP.

In maize, SUS is encoded by three genes. The first described and main source of SUS in maize endosperm cells is encoded by *Shrunken1* (*Sh1*) (Chourey and Nelson, 1976), also known as *Sus*‐*Sh1* (Zm00001d045042; McCarty *et al.*, 1986). In the *sh1* mutant, starch content and seed weight are significantly reduced, and the crown of the kernel has a shrunken, collapsed phenotype. However, in an assay of SUS activity, a significant amount of enzyme activity (8.4% of wild type, WT) is presented in a null *sh1* mutant, indicating *Sh1* could have homologous genes in the maize genome (Chourey and Nelson, 1976). This hypothesis was confirmed by characterization of a second *Sus* gene, *Sus1* (Zm00001d047253; Shaw *et al.*, 1994; McCormick *et al.*, 1982). The function of SUS1 appears to be different from that of Sh1, because Sh1 is mainly expressed in the endosperm and is essential for starch synthesis, whereas SUS1 is mainly expressed in the embryo, where it plays a role in the maintenance of cell wall integrity (Duncan *et al.*, 2006). When the double mutant of *sh1* and *sus1* was generated, SUS enzyme activity was further decreased in the endosperm, and starch content and kernel weight were lower than those of the single *sh1* mutant, indicating that Sh1 and SUS1 have functional redundancy in starch synthesis (Chourey *et al.*, 1998). The third *Sus* gene was identified in 2002; this gene was originally designated *Sus3* but was then renamed *Sus2* (Zm00001d029091; Carlson *et al.*, 2002). *Sus2* is widely expressed and can be detected in the roots, stems, leaves and grains of maize, but the levels are very low, and no specific phenotype was reported for the mutant.

It is becoming evident that the endosperm‐specific transcription factor (TF) Opaque2 (O2) functions as a central player that not only transcriptionally regulates expression of most zein storage protein‐encoding genes but also directly and indirectly regulates starch synthesis (Li *et al.*, 2015; Zhan *et al.*, 2018; Zhang *et al.*, 2016). In this study, we created a genetic screen for *o2* enhancers by ethyl methanesulfonate (EMS)‐induced mutagenesis. Because many *o2* mutants create an opaque mature kernel phenotype, a mild reduction in the synthesis of starch or storage proteins resulting from mutations in *o2* enhancers may not cause a visible change in the phenotype of *o2* seeds. A previous platform used γ‐irradiation to mutagenize a vitreous *o2* variety, quality protein maize (QPM) and created many opaque QPM variants to identify *o2* modifier genes (Chen *et al.*, 2014). However, γ‐irradiation usually causes large deletions containing many genes, which may require more genetic validation in the following analysis. Therefore, we used EMS treatment to screen *o2 e*nhancer genes that destroys the ability of the *o2* modifiers to effectively modify the *o2* phenotype. We found an opaque mutant that was later determined to encode the Sh1 protein. The *o2*;*oen1* double mutant exhibited dramatically reduced SUS enzyme activity and was severely defective in endosperm filling compared with the *o2* and *oen1‐1* single mutants. We demonstrated that O2 has significant transactivation effects on all the *Sus* genes, particularly the *Sus1* and *Sus2* promoters. We also found that although the spatial and temporal expression patterns of *Sh1*, *Sus1* and *Sus2* diverged, the three genes are functionally conserved in maize endosperm, and transactivation of *Sus1* and *Sus2* by O2 is a supplement to SUS‐mediated endosperm filling in maize.

## Results

### Screening for *o2* enhancers by EMS mutagenesis

We used EMS‐induced mutagenesis to screen *o2* enhancers with mutations that were expected to enhance the phenotype of *o2*. However, *o2* endosperm is non‐vitreous, that is opaque, in most maize backgrounds. A double mutant of *o2* and its enhancer might be indistinguishable from *o2* in terms of endosperm opacity. QPM is a vitreous version of the *o2* mutant, with genetic suppressors (*o2* modifiers) that modify the endosperm texture, creating a vitreous, hard phenotype. This mutagenesis method could identify *o2* modifier genes themselves, or unrelated genes that simply exacerbate the *o2* phenotype (*o2* enhancers). If an *o2* enhancer is mutated, endosperm modification in QPM could be disrupted and produce the opaque phenotype. Thus, we treated pollen of K0326Y, a QPM inbred line, with EMS and applied it to the associated ears. The resulting seeds were planted for self‐pollination, yielding 1500 M1 ears. One ear from this screen was found to segregate progeny kernels with normal (vitreous and plump) and mutant (opaque and shrunken) phenotypes in a 3:1 ratio (Figure S1a). To investigate whether this mutant resulted from a single‐gene mutation, mutant and normal K0326Y plants were crossed, and then, F_1_ plants were self‐pollinated. A quarter of the seeds from the resulting ears segregated with an opaque and shrunken phenotype (Figure S1b), indicating this mutation was likely a monogenic recessive mutation. It was therefore designated *o2 enhancer1* (*oen1*). The 100‐kernel weight (KW) of *oen1* in the K0326Y background (K0326Y‐*oen1*) was only 11.00 g, which was 36% lower than that of normal K0326Y (17.10 g; Figure S1c). The mutant seeds exhibited a dramatic reduction in germination (less than 6% compared to normal K0326Y), but once germinated, they developed into normal‐appearing, fertile plants, although plant height was apparently shorter than that of the normal K0326Y plants (Figure S1d). These observations imply *Oen1* plays a role in endosperm filling and has pleiotropic effects on plant development.

### Phenotypic analysis of *o2*, *oen1*, and *o2;oen1*

**Figure 1:**
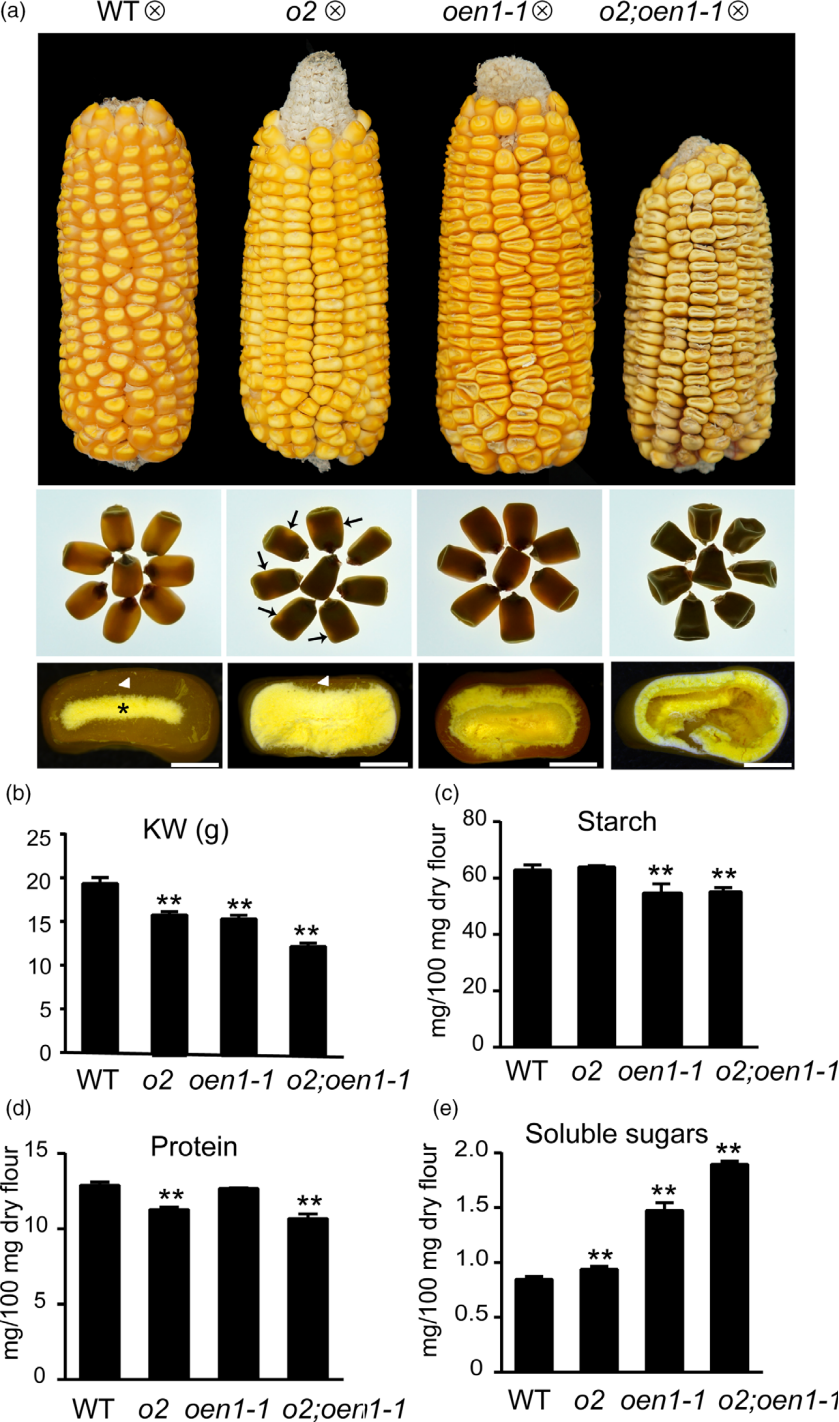
Phenotypes and biochemical analysis of WT, *o2*, *oen1‐1* and *o2;oen1‐1* in the W64A background. (a) Ear and kernel phenotypes. Top panel, ear phenotypes; middle panel, kernel vitreousness as observed on the light box. A small portion of vitreous endosperm with light translucency in *o2* seeds is indicated by arrow; bottom panel, kernel transverse sections. Arrowhead and asterisk indicate the vitreous and starchy endosperm, respectively. Scale bars, 2 mm. (b) The 100‐kernel weight of WT, *o2*, *oen1‐1* and *o2;oen1‐1*. (c) Starch content of WT, *o2*, *oen1‐1* and *o2;oen1‐1*. (d) Protein content of WT, *o2*, *oen1‐1* and *o2;oen1‐1*. (e) Levels of soluble sugars of WT, *o2*, *oen1‐1* and *o2;oen1‐1*. The data represent the mean and standard deviation (SD) of independent triplicate (d) or quadruplicate (c and e) or quintuplicate (c) measurements. g, gram. The single and double asterisks represent significant difference (Student's *t*‐test, *P* < 0.05) and extremely significant difference (Student's *t*‐test, *P* < 0.01) compared to WT, respectively.

Because QPM is homozygous for *o2*, K0326Y‐*oen1* is thus a double mutant of *o2* and *oen1*. To compare the effects of the single and double mutations on endosperm development, K0326Y‐*oen1* was recurrently backcrossed to W64A (a WT inbred line) for two generations and then self‐pollinated for two generations. In the second backcrossing, each plant was numbered and self‐pollinated, while the pollen was used for backcrossing. Approximately one quarter of the self‐pollinated ears segregated progeny seeds with four different phenotypes, that is vitreous and plump (indicative of the presence of at least one normal allele each for *O2* and *Oen1*), vitreous and shrunken (indicative of the presence of at least one normal allele for *O2* and the homozygote for *oen1*), opaque and plump (indicative of the homozygote for *o2* and the presence of at least one normal allele for *Oen1*), and opaque and shrunken (indicative of homozygotes for *o2* and *oen1*). Subsequently, seeds from the corresponding backcrossed ears were planted and should have produced equal amounts of four different genotypes (*O2/O2*;*Oen1/Oen1*, *o2/O2*;*Oen1/Oen1*, *O2/O2*;*oen1/Oen1*, *o2/O2*;*oen1/Oen1*). The WT, two single‐gene mutants and the double mutant were isolated by self‐pollinating the four different plants. The homozygous ears for each genotype resulting from one additional propagation are shown in Figure 1a.

Similar to WT and *o2*, the *oen1* and *o2*;*oen1* double mutants had normal seed sets (Figure 1a). WT seeds typically have well‐filled endosperms that are starchy in the inner region (indicated by the asterisk) and vitreous in the outer region (indicated by the arrowhead). When WT seeds were placed on a light box, light transmission was observed through the vitreous endosperm, demonstrating a harder phenotype. The *o2* endosperm was fully filled, but contained only a small amount of vitreous endosperm (indicated by the arrowhead), thereby allowing for a little light translucency (indicated by arrows). The *oen1* seeds exhibited a shrunken phenotype in the crown area, but vitreous endosperm could be formed, although it was less than WT. The double mutant seeds were dramatically shrunken and contained almost no vitreous endosperm. On a light box, the *o2*;*oen1* seeds were even more opaque than the *o2* seeds (Figure 1a). The double mutant exhibited dramatically lower grain filling than the *oen1* single mutant. The seed body of the double mutant was almost hollow, with a larger cavity in the centre of the seed. Nevertheless, embryos in *oen1* and the double mutant appeared normal. The *oen1* plant was clearly shorter than the WT and *o2* plants but taller than the double mutant (Figure S2). Since O2 is specifically expressed in the endosperm, the more reduced plant height in the double mutant might partially result from effects on growth during the germination and seedling stages, due to greater reduction in storage reserves.

To investigate the effects on endosperm filling, we measured the biochemical parameters of the WT and mutant seeds. The kernel weights (KW) of *o2* and *oen1* were 9% and 11% lower than WT. The KW of *o2;oen1* was 34% less than the WT, and was significantly lower than those of the single mutants, consistent with the largely hollowed endosperm of this mutant (Figure 1b). In *oen1* and the double mutant, starch content, based on 100 mg mature endosperm flour, was lower than WT and *o2* (Figure 1c), whereas the protein content in *o2* and the double mutant was lower than that in the WT and *oen1* (Figure 1d), indicating that *o2* and *oen1* mainly affect the synthesis of proteins and starch. SDS‐PAGE analysis revealed that although accumulation of zein proteins was not apparently affected in *oen1*, the accumulation in the double mutant was dramatically further reduced relative to the *o2* single mutant (Figure S3). A significant amount of 19‐kD α‐zeins was synthesized in *o2*, but these proteins were nearly undetectable in *o2*;*oen1* based on the SDS‐PAGE analysis, indicating that the combination of the *o2* and *oen1* mutations had synergistic effects on zein protein accumulation. Due to proteome rebalancing, the levels of the non‐zein proteins in *o2* and *o2;oen1* were compensatorily increased (Figure S3). The content of soluble sugars gradually increased in *o2*, *oen1* and the double mutant compared with WT (Figure 1e), indicating that the incorporation of sugars into starch is impaired in these mutants.

### Cloning of *Oen1*

**Figure 2:**
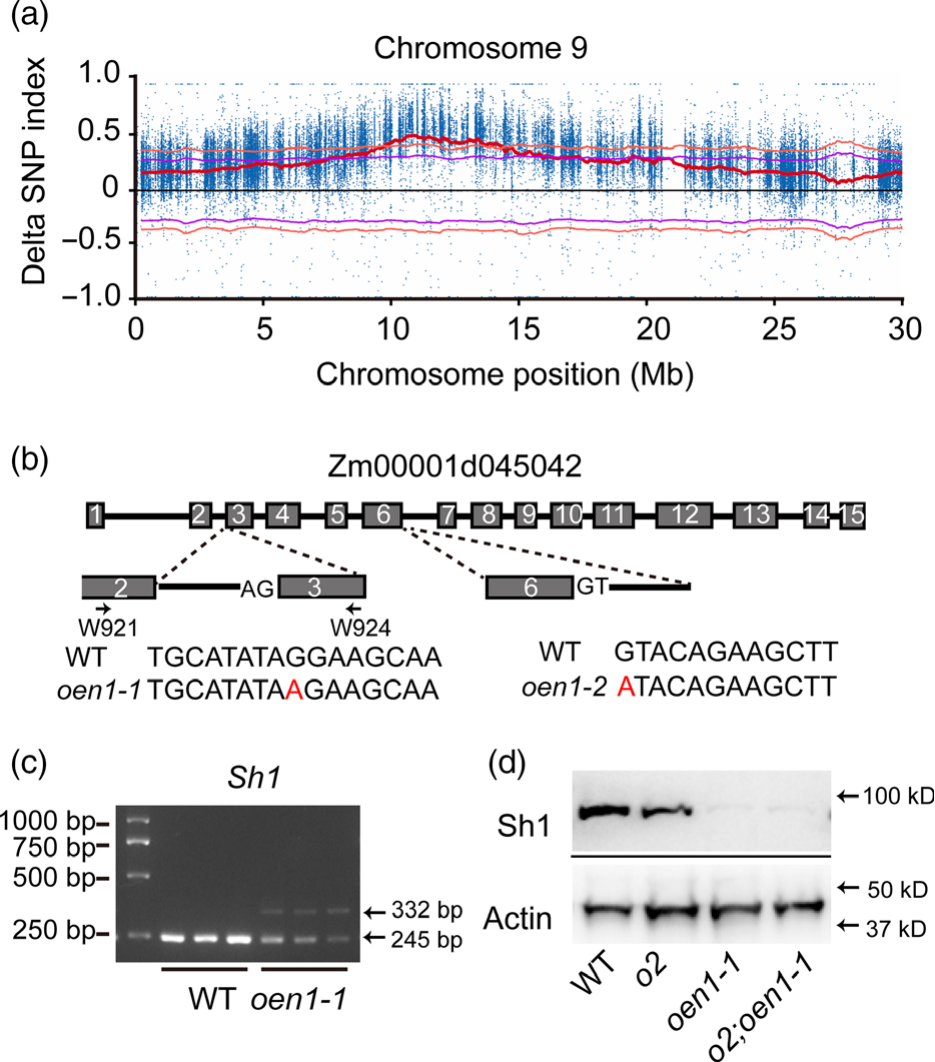
Map‐based cloning and genetic complementation test of *oen1‐1*. (a) Location of *oen1* in the 0–28 Mb region of chromosome 9, as determined by the mapping‐by‐sequencing strategy. The graph shows that SNP positions (*x*‐axis) and delta SNP‐index values (*y*‐axis, blue points) depicting the difference in SNP‐Index values between the High and Low bulks (shrunken and WT bulks). The red line is average delta SNP‐index, the purple line is delta SNP‐index 95% confidence intervals, and the orange line is delta SNP‐index 99% confidence intervals. (b) Gene structure of Zm00001d045042 (*Oen1*) and the mutant sites of *oen1‐1* and *oen1‐2*. (c) RT‐PCR detection of *Oen1* transcripts in 12‐DAP endosperms of WT and *oen1‐1*. (d) Immunoblotting analysis of Sh1 in 12‐DAP endosperms of WT *o2*, *oen1‐1* and *o2;oen1‐1*. Actin was used as an internal control.

During the introgression of *oen1* into W64A, self‐pollinated plants with the *O2/O2*;*oen1/Oen1* genotype segregated plump and shrunken seeds at a ratio of 3 : 1 (Figure S4a and b). A total of 150 seeds of each phenotype from BC_1_F_2_ ears were germinated for DNA extraction. We performed whole‐genome sequencing of the two DNA pools and the two parents K0326Y and W64A (See in Materials and Methods). Comparison of the single‐nucleotide polymorphism (SNP) index between the mutant and WT pools revealed a 28‐Mb region, containing nine candidate genes, near the short arm of chromosome 9 (Figure 2a). Genomic sequencing of *oen1* mutants revealed a G‐to‐A mutation at the splice acceptor site in the second intron (the last nucleotide) of Zm00001d045042, which could result in defective RNA splicing of this intron (Figure 2b). We designed a primer pair (W921 and W924) flanking the second and third exons of Zm00001d045042 and performed RT‐PCR to verify the RNA splicing defect using cDNAs from 12‐DAP old endosperms. The WT only gave rise to the correct band size (245 bp), whereas the mutant produced two bands, one being the spliced band at a significantly reduced level and the other being the unspliced (332 bp).

Zm00001d045042 known as *Sh1*, encodes a SUS that catalyses the cleavage of sucrose to form fructose and UDP‐glucose. Immunoblotting showed that accumulation of the Sh1 protein was reduced to a level that was barely detectable in *oen1* and *o2;oen1* (Figure 2d). Mutations in *Sh1* result in a shrunken kernel phenotype. To genetically confirm that this mutation was responsible for the *oen1* phenotype, an allelic test between *oen1* and *sh1‐ref* was performed. The cross of the two mutants failed to exhibit complementation in the F_1_ hybrids, confirming that *oen1* is allelic to *sh1*‐*ref* (Figure S5). To further verify the allelism and action of *sh1* as an *o2* enhancer, W64A*o2* was pollinated with EMS‐treated W64A pollen, and the resulting seeds were planted and self‐pollinated. We screened an ear that segregated progeny seeds with an enhanced *o2* phenotype (Figure S6a). The appearance of four different seed phenotypes (vitreous and plump, vitreous and shrunken, opaque and plump, and opaque and shrunken) on the ear was similar to that shown in Figure 1a. We identified a G‐to‐A mutation at the splice acceptor site in the sixth intron (the first nucleotide) of Zm00001d045042, which results in defective RNA splicing, as observed for *oen1* (Figure 2b). A complementation test revealed that *oen1* and the new mutant were allelic (Figure S5); thus, these alleles were referred to as *oen1‐1* and *oen1‐2* in subsequent studies. The alterations in KWs and levels of starch, soluble sugar and total proteins and the accumulation patterns of zein and non‐zein proteins in *oen1‐2* and *o2*;*oen1‐2* correlated well with those in *oen1‐1* and *o2*;*oen1‐1* (Figures S3, S6 and S7).

### Synergistic reduction in SUS activity in the *o2*;*oen1* double mutant

**Figure 3:**
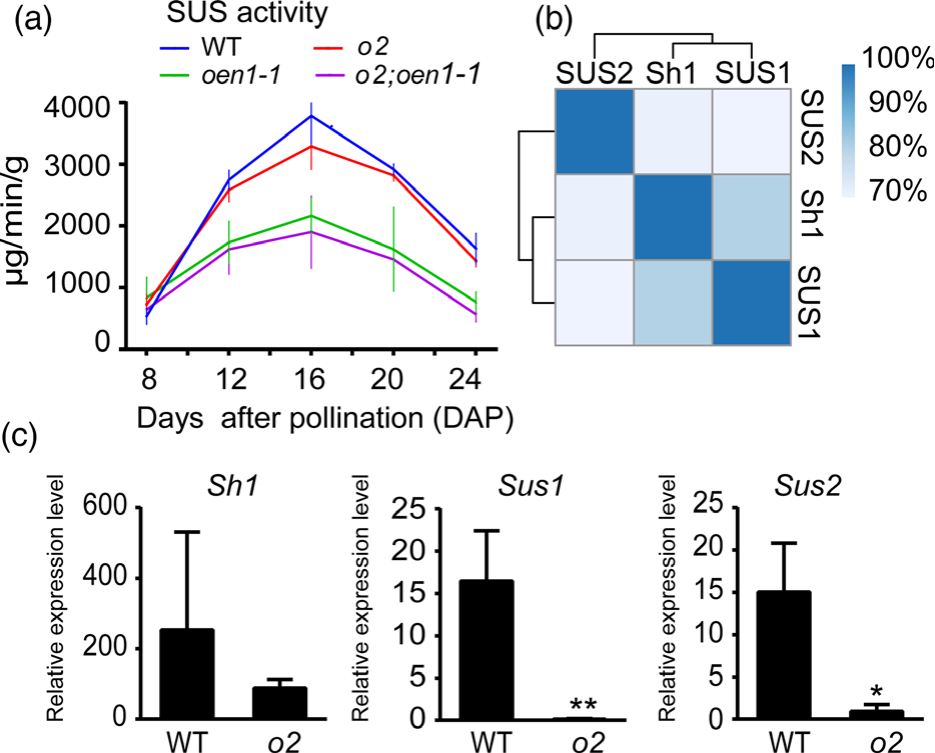
Total SUS activity and transcript levels of the three *Sus* genes in the developing endosperms of WT, *o2*, *oen1‐1*, and *o2;oen1‐1*. (a) The total SUS activity in the developing endosperms of WT, *o2*, *oen1‐1*, and *o2;oen1‐1* from 8 to 24 DAP. The data at each time point represent the mean ± SD of six measurements except for 8 DAP (quadruplicate measurements). The production of 1 μg of sucrose in 1 min per gram of tissue is defined as one unit of SUS activity (μg/min/g). (b) Amino acid sequence identity of the three SUS enzymes. (c) Transcript levels of the three *Sus* genes in WT and *o2* endosperms at 12 DAP. All expression levels are normalized to that of *Ubiquitin*. The data represent the mean ± SD of triplicate measurements. The single and double asterisks represent significant difference (Student's *t*‐test, *P* < 0.05) and extremely significant difference (Student's *t*‐test, *P* < 0.01) compared to WT, respectively.

The double mutant of *o2* and *oen1* manifested more than the simple combination of the non‐vitreous and collapsed crown phenotypes. The *o2*;*oen1 *seeds formed a much larger endosperm cavity than *oen1* (Figure 1a) and accumulated significantly less zein proteins than *o2* (Figure S3), indicating that SUS and the O2 TF are functionally connected by an as yet unknown biochemical mechanism. To investigate this, we measured the total SUS enzyme activity in developing endosperms of the four genotypes at 10 and 14 days after pollination (DAP). At 10 DAP, SUS activity was greatly reduced in *oen1* and the double mutant; however, no apparent change was observed in the *o2* mutant (Figure S8), probably because expression of the *O2* gene begins in the endosperm at 10 DAP, and the presence and absence of *O2* have no effect on SUS enzyme activity. By 14 DAP, when storage reserves are actively synthesized in the endosperm, SUS activity in the *o2* mutant was observed to be significantly reduced compared with WT, although SUS activity in the *oen1* mutant was even lower. In the double mutant, the activity was even more reduced (Figure S8). To verify that the *o2* mutation affects SUS activity, we repeated the experiment the following year with additional time points during endosperm development (Figure 3a). From 8‐24 DAP, SUS activity in WT was generally highest among the four genotypes, while the activity in *o2* was slightly lower than WT. However, SUS activity was greatly reduced in *oen1* and more so in *o2;oen1* (Figure 3a). The *Sh1* gene is highly expressed in the endosperm and contributes maximum SUS activity in this storage organ. The results of the enzyme activity assays conducted in the two years indicated that O2 could be a regulator of SUS activity.

We measured the levels of sucrose, fructose and glucose in the kernel by ion chromatography. The varying contents of these sugars in the mutants did not show a trend that was associated with alterations in SUS activity during endosperm filling (Figure S9), suggesting that levels of these metabolites are regulated by a complex enzyme network.

### O2 transcriptionally regulates *Sus* genes

Three genes in maize encode SUS, that is *Sh1*, *Sus1* and *Sus2*. These proteins are evolutionarily conserved and share high amino acid sequence identity. The homology between Sh1 and SUS1 is 80%, that between Sh1 and SUS2 is 71%, and that between SUS1 and SUS2 is 70% (Figure 3b and Figure S10). Although the *Sus* genes exhibit spatially different expression patterns, they are all expressed in the endosperm, with *Sh1* exhibiting the highest and *Sus2* exhibiting the lowest transcript accumulation. We performed quantitative RT‐PCR (RT‐qPCR) to analyse the expression of *Sus* genes in 12‐DAP endosperms of the WT and *o2*. As shown in Figure 3c, the transcript levels of *Sus1* and *Sus2* were dramatically reduced to a barely detectable level in *o2* compared with WT. Although expression of *Sh1* was downregulated in *o2*, the fold changes were not comparable to those for *Sus1* and *Sus2*, consistent with our previous published RNA‐seq data (Zhang *et al.*, 2016).

**Figure 4:**
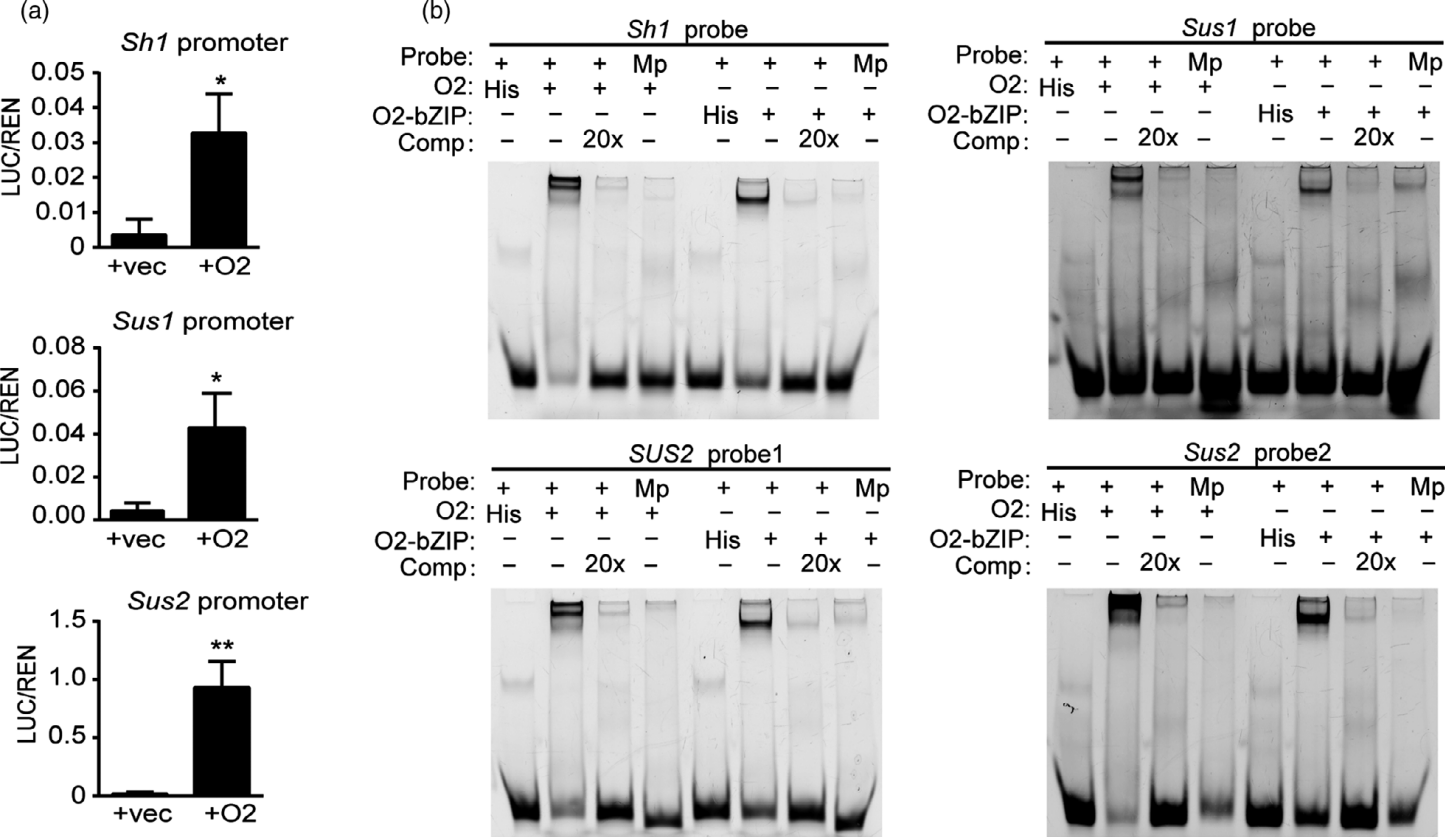
Transactivation assay and EMSA of the *Sus* gene promoters with O2 and O2‐bZIP proteins. (a) Dual‐luciferase assays of the promoters of *Sh1*, *Sus1* and *Sus2* with O2 in Arabidopsis protoplasts. REN, Renilla luciferase; LUC, firefly luciferase. LUC/REN, ratio of LUC activity to REN activity. The data represent the means ± SDs of three or more independent replicates. The single and double asterisks represent significant difference (Student's *t*‐test, *P* < 0.05) and extremely significant difference (Student's *t*‐test, *P* < 0.01) compared to WT, respectively. (b) EMSA of ACGT‐containing probes in the three *Sus* promoters with His‐O2 and His‐O2‐bZIP proteins. Comp, competing probes not labelled with biotin; Mp, mutated probes with a mutation in the corresponding ACGT core element. The promoter and the probe sequences of the three *Sus* genes are in Appendix S1.

To investigate whether O2 directly transactivates the transcription of the three *Sus* genes, a dual‐luciferase transcriptional activity assay was performed. In this system, the Renilla LUC (REN) reporter gene driven by the cauliflower mosaic virus 35S promoter was used as an internal control, and the *Sus* gene promoters were fused with the firefly luciferase coding sequence, yielding the reporter vectors P*Sh1*‐LUC, P*Sus1*‐LUC and P*Sus2*‐LUC. The effector plasmid was generated by fusing the coding region of *O2* to the 35S promoter (35S‐O2). Coexpression of P*Sh1*‐LUC, P*Sus1*‐LUC or P*Sus2*‐LUC with 35S‐O2 all resulted in a significant increase in LUC activity. The *Sus2* promoter exhibited the strongest activation by O2 (100‐fold), followed by the *Sus1* promoter (20‐fold), whereas the *Sh1* promoter exhibited only a 10‐fold increase (Figure 4a), consistent with the transcript level of this gene being the least reduced in the *o2* mutant (Figure 3c).

Evidence from previous ChIP‐seq data showed that O2 could bind to the *Sus1* promoter and potentially regulate its expression (Zhan *et al.*, 2018). We performed an electrophoretic mobility shift assay (EMSA) to confirm the direct binding of the *Sus* promoters by O2. O2 belongs to the bZIP TF family, members of which recognize a motif containing the ACGT core element (O2 box). *Sh1* and *Sus1* genes were found to bear one O2 box within 800‐bp promoter regions, and *Sus2* had two such motifs. Oligonucleotides (50‐60 bp) labelled with FAM fluorescent molecules were used as probes to examine the binding affinity. Binding of the His‐O2 fusion protein and the His‐O2‐bZIP(aa^229‐293^) fusion protein to the probes could be visualized as retarded bands in the gel. The results showed that O2 and the O2‐bZIP domain protein could specifically bind to the probes from all *Sus* gene promoters (Figure 4b). When the O2 box in the four probes was mutated, the retarded bands were nearly abolished. The binding specificity was verified by a competition experiment, in which addition of unlabelled intact probes in the reaction resulted in loss of all the retarded bands (Figure 4b). Because the two O2 boxes in the *Sus2* promoter were recognized by O2, both boxes must be functional. Collectively, these results demonstrate that O2 can regulate all these *Sus* genes, although the apparent strengths of transactivation differ.

### Phenotypes of *sus* gene mutants

**Figure 5:**
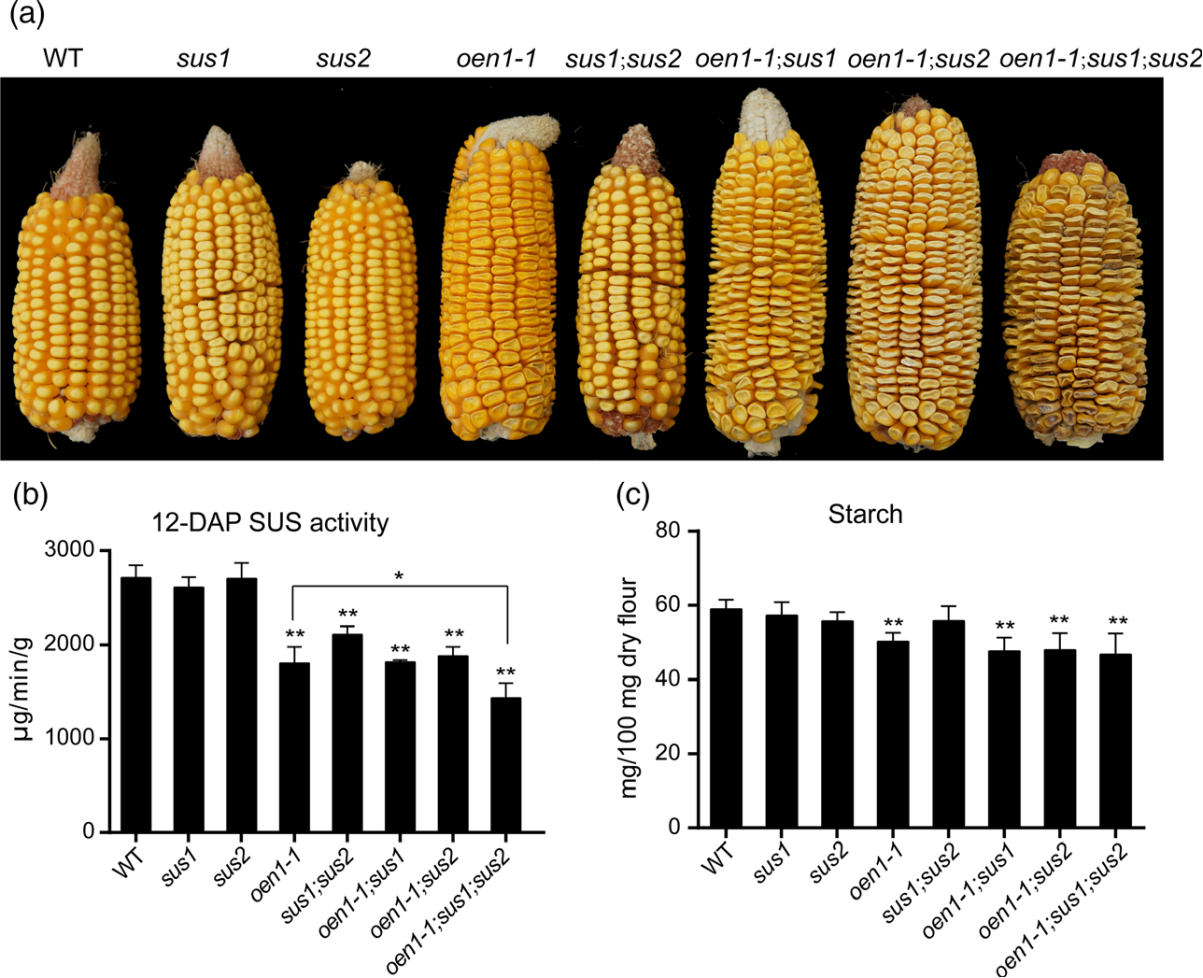
Phenotypes of WT and single, double and triple mutants of the three *sus* genes. (a) Ears phenotypes of all genotypes of the three *sus* genes. (b) The total SUS activity of all genotypes from 12‐DAP developing endosperms. The data at each time point represent the mean ± SD of six measurements. The production of 1 μg of sucrose in 1 min per gram of tissue is defined as one unit of SUS activity (μg/min/g). The single and double asterisks represent significant difference (Student's *t*‐test, *P* < 0.05) and extremely significant difference (Student's *t*‐test, *P* < 0.01) compared to WT or *oen1‐1*, respectively. (c) Starch content of all genotypes in 100 mg dry endosperm flour. The data represent the mean ± SD of quadruplicate measurements. The double asterisks represent significant difference (Student's *t*‐test, *P* < 0.05) compared to WT.

To investigate whether the larger seed cavity in the *o2*;*oen1* double mutant can be partially explained by the markedly reduced expression of *Sus1* and *Sus2* and the absence of *Sh1*, we generated different combinations of *sus* mutants (Figure 5a). We obtained the *sus1* and *sus2* mutants from the Maize EMS‐induced Mutant Database (MEMD; http://www.elabcaas.cn/memd/); each of these mutants harbour a stop codon in the coding region. Consistent with previous reports (Carlson *et al.*, 2002; Chourey *et al.*, 1998; Shaw *et al.*, 1994), *sus1* and *sus2* did not show any apparent phenotype in the endosperm. The double mutant of *sus1* and *sus2* also appeared normal, similar to the WT. *Sh1* and *Sus1* are located on the long and short arms, respectively, of chromosome 9. The double mutant of *sh1* (using *oen1‐1*) and *sus1* was created by screening their recombinants. As shown in Figure S11, combination of *sh1* and *sus1* or *sus2* resulted in the seed cavity being dramatically enlarged compared with that of the *sh1* single mutant. This enlargement was further enhanced in the triple mutant, which exhibited the largest hollow seed compartment among all the mutants. Similar to *o2*;*oen1*, the *sh1*;*sus1* and *sh1*;*sus2* double mutants and the *sh1*;*sus1*;*su2* triple mutant had full seed sets on the ears. Due to severely insufficient endosperm filling, seeds in the central cob of these plants were all non‐vitreous and extremely shrunken, forming flattened kernels during seed desiccation (Figure S11).

We measured SUS activity in 12‐DAP endosperms and starch content in mature endosperms of all combinations (Figure 5b and c). Although SUS activity was not apparently affected in single mutants of *sus1* and *sus2*, it was significantly reduced in their double mutant. In the triple mutant, SUS activity was more reduced compared to the single mutant *oen1‐1* (Figure 5b). Consistent with Sh1 being a major contributor of SUS activity, starch content was only significantly reduced in *oen1‐1* and combinations containing this mutant gene (Figure 5c). We also measured sucrose, fructose and glucose contents. Consistent with the observation in Figure S9, no obvious trend of sucrose, fructose and glucose contents of all genotypes was observed in 12‐DAP endosperms (Figure S12).

## Discussion

### Transcriptional regulation of *Sus* genes by O2

Grain starch and protein synthesis, which is regulated by a few common TFs (Zhang *et al.*, 2019; Zhang *et al.*, 2016), are coordinated during endosperm development. O2 was initially identified as a transcriptional regulator of 22‐kD α‐zein genes (Schmidt *et al.*, 1992), but was later found to regulate a large number of genes by experimental (Gallusci *et al.*, 1996; Kemper *et al.*, 1999; Lohmer *et al.*, 1991; Maddaloni *et al.*, 1996) and genomic (Hunter *et al.*, 2002; Li *et al.*, 2015; Zhan *et al.*, 2018) studies. The nutritional quality and yield traits, in terms of the synthesis of zein proteins and starch, are coordinately regulated by O2, which directly transactivates the expression of *PPDKs* and *SSIII* with another endosperm‐specific TF, PBF1 (Zhang *et al.*, 2016). Based on these findings, O2 clearly functions as a central factor for storage reserve synthesis; however, the complexity of the O2‐regulated network is not fully understood.

SUS activity is affected by many factors, including the enzyme level, pH of the enzyme environment, the concentration of sucrose and glucose, the phosphorylation status of the enzyme, kernel oxygen content, and assembly of the enzyme subunits (Duncan *et al.*, 2006; Duncan and Huber, 2007; Subbaiah *et al.*, 2006; Zeng *et al.*, 1998). However, the mechanism of transcriptional regulation of the *Sus* genes is unknown. Based on published RNA‐seq data (Chen *et al.*, 2014), the three genes are transcribed in all studied tissues. The high expression of *Sh1* and the striking endosperm phenotype of the mutant of this gene suggest an important role for Sh1 in endosperm filling (Figure 3c and Figure S11). Because mutants of *sus1* and *sus2* exhibit no apparent phenotype (Figure S11) and low transcript accumulation in the endosperm, these genes were previously thought not to have an essential role in endosperm filling. The divergence in the biological roles of the three *Sus* genes probably results from difference in transcript abundance, rather than enzyme function. Indeed, the first intron of *Sh1* is one of the strongest enhancers of plant gene expression. A 145‐bp fragment from the 1028‐bp *Sh1* intron has the capacity to increase gene expression by 20‐ and 50‐fold when introduced into the 5′ region of a gene or construct (Clancy and Hannah, 2002; Vasil *et al.*, 1989). This intron‐mediated enhancement should be post‐ or cotranscriptional; however, the transcriptional regulation of *Sh1* and the other two *Sus* genes has not been elucidated.

A key function of SUS in maize endosperm is to process sucrose for starch synthesis in an energy‐conserving manner, relative to CWI‐mediated sucrose cleavage (Koch, 2004). The *o2*;*oen1* double mutant exhibited considerable defect in endosperm filling and a significant reduction in SUS activity, compared with the *o2* single mutant, indicating a potential role for O2 in the transcriptional regulation of *Sus* genes. We observed a greater reduction in starch content and the accumulation of α‐zeins in the double mutant of *o2* and *oen1* compared to the *o2* single mutant (Figure 1c, Figures S3 and S7), suggesting a synergistic or additive action of O2 and Oen1 on starch and protein synthesis and *oen1* acting as an enhancer for *o2*. On the one hand, mutation in *Oen1* causes a certain loss of SUS activity, and adding *o2* to that reduces expression of *Sus1* and *Sus2*. This results in the total SUS activity being reduced even further and thus a synergism being arisen in terms of the extent of endosperm filling. On the other hand, this could be also explained by additive effects between the two unrelated processes. The *o2* effect on protein bodies through zein expression and the *sh1* effect on starch content could be independent. They combine to give a more severe kernel defect through the two affected processes. For protein synthesis, most amino acids derive from the transformation of intermediate products from the tricarboxylic acid cycle and pentose phosphate pathway in vivo (Munoz‐Bertomeu *et al.*, 2009). The starting substrates of these biological reactions are mainly provided by sucrose cleavage. Therefore, the synthesis of the most abundant α‐zein proteins in the endosperm is not only regulated at the transcriptional level by O2, but is also affected by the availability of amino acids and thus the SUS activity. However, it could be possible that *o2* causes a high degree of α‐zein reduction and *sh1* deficiency exacerbates this effect by an unknown mechanism.

Within their 800‐bp promoter regions, the three genes were found to contain at least one O2 box that could be specifically bound and transactivated (Figure 4b). This indicates that *Sus* genes have a common regulatory network. We determined that O2 exhibits much stronger transactivation effects on the *Sus1* and *Sus2* promoters than on the *Sh1* promoter, consistent with the expression of *Sh1* being least affected in *o2* relative to that of *Sus1* and *Sus2* (Figure 3c). The dominant expression of *Sh1* among the three *Sus* genes is probably conferred by the enhancer located in the first intron of this gene or by other as yet unknown factors, such as the low‐oxygen condition in the endosperm (McElfresh and Chourey, 1988; Ricard *et al.*, 1998). Nevertheless, the hollow endosperm phenotype of the *o2*;*oen1* double mutant implies that O2‐mediated transactivation of *Sus1* and *Sus2* is an essential supplement to *Sh1* expression. To test this hypothesis, we created different combinations of *sus* mutants and demonstrated that stacking of *oen1‐1* with either *sus1* or *sus2* caused greater enlargement of the seed cavity. The *oen1‐1*;*sus1*;*sus2* triple mutant exhibited the most severe defects in endosperm filling (Figure 5a), indicating that SUS drives endosperm filling in a dose‐dependent manner (Figure 5b). We noted that SUS activity was more greatly reduced in the *oen1‐1*;*sus1*;*sus2* triple mutants compared to the single *oen1* mutant (Figure 5b), but the starch content failed to show this trend (Figure 5c). This is probably because the starch content was calculated by measuring the amount of starch in 100 mg of endosperm flour rather than per single seed.

### Roles of SUS in maize endosperm filling and plant development

The lack of SUS activity in the *oen1‐1*;*sus1*;*sus2* triple mutant resulted in dramatic reduction in endosperm mass. In spite of reduced invertase activity in the *mn1* mutant (Cheng *et al.*, 1996; Miller and Chourey, 1992), significant amounts of storage reserves continue to be synthesized in the endosperm, implying other mechanisms influence sucrose transport into the kernel. This raises questions regarding interactions between the *Mn1* and *Sus* gene expression. We propose two hypotheses to explain the functional relationship between the *Mn1* and *Sus* genes: (i) CWI2 and SUS function complementarily in a parallel way. Each of these genes alone is insufficient for efficient sucrose cleavage and effective endosperm filling; (ii) CWI2 and SUS work sequentially to influence endosperm development and filling. Both outcomes support the idea that the products of the two enzymes contribute to endosperm filling, but differ in location and reaction mechanisms and outcomes. The CWI2 pathway for sucrose cleavage produces twice as much hexose (fructose and glucose) as the SUS pathway (fructose and UDP‐glucose). Both *mn1* and the *sus* triple mutant manifest dramatic reductions in endosperm size and weight, but *mn1* exhibits additional developmental defects at the BETL at a relatively early stage. It is generally accepted that glucose creates signals that trigger BETL cell division and differentiation (Borisjuk *et al.*, 2004; Zheng and Wang, 2010) and the limited supply of hexoses in the *mn1* mutant might be a reason for the abnormal effect at the BETL (Cheng *et al.*, 1996; Kang *et al.*, 2009; Miller and Chourey, 1992). *Mn1* is predominantly expressed between 6 and 8 DAP, coincident with the mitotic division and differentiation of endosperm cells, whereas the major SUS‐encoding gene, *Sh1*, is highly expressed after 10 DAP, when endosperm filling begins (Chen *et al.*, 2014). Based on these temporal differences, one could envision CWI2 mediates BETL development, while SUS mediates sucrose cleavage after it is imported through the BETL. In addition to starch synthesis, UDP‐glucose is also used to synthesize cell wall polysaccharides (Albrecht and Mustroph, 2003; Doblin *et al.*, 2002; Ruan *et al.*, 2003). A deficiency in SUS may result in reduced plant height by affecting cell wall formation (Figures S1d and S2).

## Conclusions

**Figure 6:**
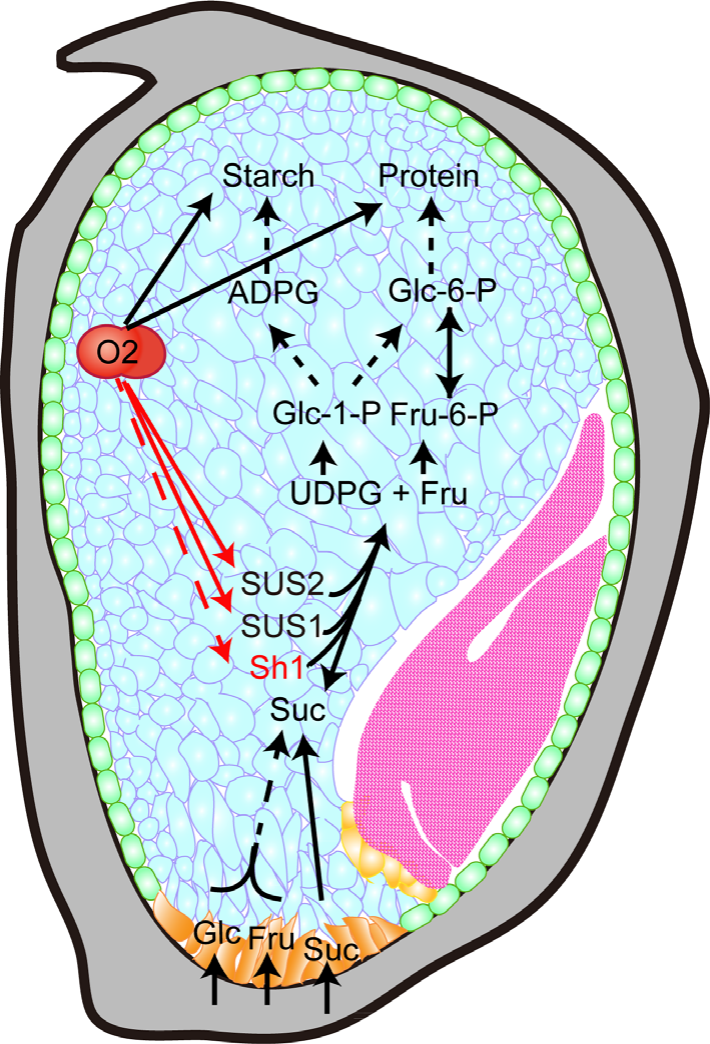
A proposed model for the transcriptional regulation of the three *Sus* genes by O2.

Endosperm filling determines seed size and weight and is dependent on the efficient transport of soluble sugars and amino acids into developing seeds for the synthesis of starch and storage proteins. After 10 DAP, sugar import into maize kernels occurs at a high rate, with two enzymatic pathways that facilitate their process. To efficiently process sucrose, transcription of *Sus1* and *Sus2* by the endosperm‐specific transcription factor O2 could increase the expression of SUS. Because the synthesis of starch and storage proteins is initiated at 10 DAP in starchy endosperm cells, the regulation of their synthesis must be spatially and temporally coordinated, which is critical for the formation of the vitreous and plump endosperm. O2 functions to network the regulation of protein and starch synthesis by transactivation of most of zein gens, *PPDKs* and *SSIII*. Our work identifies O2 as a central transcriptional regulator that coordinates different processes of endosperm filling via genes involved in sucrose cleavage, starch synthesis and expression of storage protein genes (Figure 6).

## Experimental procedures

### Genetic materials

The QPM line K0326Y is an *o2* mutant (Holding *et al.*, 2011). The *o2* modifiers modify its opaque endosperm phenotype to a vitreous version. The K0326Y‐*oen1* mutant was created by EMS‐induced mutagenesis of K0326Y, thereby K0326Y‐*oen1* being a double mutant of *o2* and *oen1*. The single *oen1* mutant (*oen1‐1*) was isolated by recurrently backcrossing to W64A (a WT inbred line) for two generations. The homozygous *oen1‐1* ears were obtained by self‐pollination of BC_2_ seeds for two generations.

An allelism test was carried out by gene complementation in the F_1_ hybrids of *oen1* and *sh1*‐*ref*. The *sh1*‐*ref* seeds were generously provided by Professor Yubin Li from the Biotechnology Research Institute, Chinese Academy of Agricultural Sciences. To further verify the action of *sh1* as an *o2* enhancer, another mutant that enhances the *o2* phenotype was created by EMS mutagenesis. EMS‐treated pollen of W64A was applied to the silks of W64A*o2* ears. The resulting seeds were planted for self‐pollination to screen for ears that segregated progeny with an enhanced *o2* phenotype. Due to the allelism of *oen1* and the new mutant, these alleles were named *oen1‐1* and *oen1‐2*, respectively.

### Measurement of the soluble sugar, starch and protein content in mature dry seeds

A dozen mature kernels of each genotype were ground to fine powder using a tissue grinder for the analysis of the soluble sugar, starch and protein content. All measurements for each genotype were performed in at least triplicate.

For the measurement of soluble sugar content, 100 mg of mature kernel flour was weighed and added into 80% (vol/vol) ethanol to extract soluble sugars. The anthrone method for determining soluble sugar content was previously described (Wang *et al.*, 2013).

The starch content per endosperm was determined by the Megazyme Total Starch Assay Kit (catalog number: K‐TSTA‐50A) based on the activities of thermostable α‐amylase and amyloglucosidase. Each sample was measured four times.

The total protein content in the mature seed flour was determined with using a Dumas rapid nitrogen determination analyser (rapid N exceed N/protein analyser, Elementar, Germany). Approximately 50 mg of seed flour was weighed into tin boats without pretreatment and pressed to pellets using a manual pressing tool. The nitrogen content was then detected by a thermal conductivity detector. Analyses were run using a standard method implemented in the instrument software, with a total analysis time of approximately 5 min. A protein factor of 6.25 was applied to calculate the average protein content. Each sample was measured three times.

### Mapping‐by‐sequencing of *oen1*

The BC_1_F_2_ ears of K0326Y‐*oen1* and W64A with the segregating seeds of WT and *oen1* were used to clone the *oen1* gene by the mapping‐by‐sequencing approach. 150 seeds of each phenotype in the BC_1_F_2_ ears, and 30 seeds of each parent, K0326Y‐*oen1* and W64A, were selected and germinated. The resulting seedlings were used individually to extract genomic DNA. The individual genomic DNA for each phenotype/parent was pooled equally. Four pooled libraries with insert sizes of approximately 350–500 bp were prepared and sequenced on a HiSeq X™ Ten (Illumina) lane using 150‐base paired‐end sequencing. To identify the mutation site, the paired ‐end short reads obtained from the four samples were aligned to the reference genome of *Zea mays* (AGPv4.35) using BWA‐MEM software (Li and Durbin, 2009). SAMtools (Li and Durbin, 2009) was used to convert alignment files to BAM format and applied to SNP calling. SNP positions with SNP quality score <20 and read depth <4 were excluded, as these positions may represent spurious SNPs called due to sequencing and/or alignment errors. Then, homozygous SNPs with different genotype in parents were used, we defined the proportion of reads harbouring shrunken mutant genotype in the total short reads cover the SNP site as the SNP‐index, and Δ(SNP‐index) (Takagi et al., 2013) was calculated by subtracting the SNP‐index of the low bulk pool (WT pool) from that of the high bulk pool (shrunken pool). The average △(SNP‐index) of these two offspring bulks was calculated in a 1‐Mb interval with a 10‐kb sliding window to identify candidate regions associated with the trait. 95% and 99% confidence intervals of Δ(SNP‐index) were generated for each read depth according to Takagi et al. (2013).

The *oen1* gene was mapped in a 28‐Mb region, containing nine candidate genes, near the short arm of chromosome 9. The causal gene Zm00001d045042 was screened out by Sanger sequencing of the genomic DNA of the nine candidate genes from K0326Y and W64A.

### Zein and non‐zein extraction and SDS‐PAGE

Three kernels were taken from each genotype, and the embryos were removed. The endosperms were ground to a powder in a steel pot in a tissue grinder, and 50 mg of each sample was used for zein extraction with 0.5 mL of zein extraction buffer (3.75 mm sodium borate, 2% 2‐mercaptoethanol [v/v], 0.3% SDS and 70% ethanol; pH 10) in a 2‐mL tube at room temperature for more than 2 h. The mixture was centrifuged at 17370 *g* for 10 min, and 100 μL of the supernatant was transferred to a new tube and mixed with 10 μL of 10% SDS. A Concentrator Plus (Eppendorf) was used to dry the solution, and the material was then redissolved with 100 μL of ddH_2_O. The precipitate was extracted prior to zein buffer three additional times for non‐zein extraction. The precipitate was dried in the Concentrator Plus and vortexed with 0.5 mL of non‐zein extraction buffer (12.5 mm sodium borate, 2% 2‐mercaptoethanol [v/v] and 5% SDS) at room temperature for more than 2 h. After centrifugation at 17370 *g* for 10 min, the supernatant containing non‐zein proteins was transferred to a new tube. Then, 3 μL of the zein and non‐zein proteins was analysed by SDS‐PAGE (15%).

### Western blotting

A peptide antibody for Sh1 was prepared by Abclonal, China, in rabbits. The specific antibodies were produced using sequences unique to maize Sh1 with the following sequence: (^49^EFDALFDSDKEKYAP^63^). Total protein in the four 12‐DAP endosperm samples was extracted as described above and separated by SDS‐PAGE (4%–20%). The proteins were electrophoretically transferred to a Bio‐Rad PVDF membrane that was activated by methanol. The membrane was processed according to the manufacturer's instructions, and the *Sh1* protein was detected by the Tanon‐5200 system. The dilutions of the antibodies against Sh1 and ACTIN (Abclonal) were 1:1000 and 1:8000, respectively.

### RNA extraction, RT‐qPCR and RT‐PCR

All materials for RNA extraction were kept at −80 °C until use. For total RNA extraction of developing seeds and endosperms, samples were placed in a 2.0‐mL microfuge tube with 200 μL of RNA extraction buffer containing 150 mm LiCl, 50 mm Tris (pH 8.0), 1% SDS and 5 mm EDTA (pH 8.0). The samples were rapidly homogenized using a tissue grinder and subsequently extracted twice using an equal volume of phenol–chloroform (pH 4.2) and an equal volume of chloroform once. The aqueous phase was transferred to TRIzol (Invitrogen) for further extraction of total RNA, followed by RNA purification using the RNeasy Mini Kit (Qiagen). A total of 5 μg of total RNA from each sample were used for reverse transcription with the Superscript III First Strand Kit (Invitrogen). RT‐qPCR was performed with SYBR Green (Takara) on a Bio‐Rad CFX‐96 thermocycler. The relative expression of these genes was calculated using the ΔΔCt method. The internal control was the maize *Ubiquitin* gene. All primers used in this experiment are listed in Table S1.

### Determination of sucrose synthase activity in maize endosperms

Endosperms separated from fresh developing seeds were rapidly frozen in liquid nitrogen and kept at −80 °C until use. More than 20 endosperms were immersed in a steel tank cooled with liquid nitrogen and rapidly ground to a powder in a tissue grinder. 50 mg of endosperm powder was immediately placed into a tube cooled with liquid nitrogen to measure SUS activity. The Solarbio Sucrose Synthetase (SS) Assay Kit (visible spectrophotometry; BC0580) was used for activity determination. Sucrose synthetase catalyses the conversion of fructose and UDPG to UDP and sucrose. The reaction between sucrose and resorcin results in a colour change, with a characteristic absorption peak at 480 nm, and the enzyme activity is proportional to the colour intensity. Each sample was measured six times. We used ultraviolet visible photometer (Beckman Coulter DU730) to detect the characteristic absorption peak of 480 nm. Each sample was measured six parallel experiments.

### Determination of sucrose, glucose, fructose levels in maize endosperms

Fifty mg of freshly endosperm prepared powder was extracted with 2 mL ddH_2_O with shaking incubation for 30 min on ice. The supernatant was then moved to a new centrifuge tube after centrifugation at 13 000 rpm for 10 min at 4 °C and filtered with 0.22 μm filter. The filtered liquid was diluted with ddH_2_O by 10 fold for measurement by the ion chromatography (ICS5000, Thermo Fisher Scientific) using a CarboPac PA‐20 column (Nagamine and Komae, 1996). The mobile phase was composed of solvent A (dd H_2_O) and solvent B (200 mm NaOH).

### Dual‐luciferase assay

Isolation of Arabidopsis mesophyll protoplasts, PEG–calcium transfection of plasmid DNA, and protoplast cultivation were performed as described previously (Zhang *et al.*, 2015). The effector vector pRI101 was used for expression of *O2* driven by the 35S promoter. The reporter vector pGreenII 0800‐LUC was used to detect the transactivation of the three *Sus* gene promoters. The LUC/REN activity ratio was measured using the DLR assay system (Promega). Primers are listed in Table S1.

### Electrophoretic mobility shift assay

The expression and purification of recombinant proteins of His‐O2 and His‐O2‐bZIP(aa^229‐293^) and the EMSA procedure have been described previously (Zhang *et al.*, 2015). The fluorescently labelled DNA on the gel was detected with a Starion FLA‐9000 instrument (FujiFilm, Japan).

### Statistical analysis

Microsoft Excel (2016) was used to calculate *P*‐value via Student's t tests. The original data and details for statistical analysis were shown in the Supplemental dataset file.

## Conflict interests

The authors have no conflict of interest to declare.

## Author contributions

YW conceived the idea and supervised the project overall. YD and YW wrote and revised the manuscript together. YD designed and performed the experiments. JW and ZZ participated in the experimental design, phenotype measurements and manuscript revision.

## Supporting information


**Figure S1** The K0326Y‐oen1 mutant generated by EMS‐induced mutagenesis of K0326Y.
**Figure S2** The plant phenotype of WT, o2, oen1‐1, o2;oen1‐1 in the W64A background.
**Figure S3** SDS‐PAGE analysis of zein (upper) and non‐zein (lower) proteins of mature kernels of WT, o2, oen1‐1, o2;oen1‐1 in the W64A backcross population.
**Figure S4** The phenotype of oen1‐1 in the W64A background.
**Figure S5** Genetic complementation test of oen1‐1 with sh1‐ref and oen1‐2 alleles.
**Figure S6** Kernel phenotypes of WT, o2, oen1‐2, o2;oen1‐2 in the W64A background.
**Figure S7** SDS‐PAGE analysis of zein (upper) and non‐zein (lower) proteins of mature seeds of WT, o2, oen1‐2, o2;oen1‐2 in the W64A background.
**Figure S8** SUS activity in developing endosperms of WT, o2, oen1‐1, o2;oen1‐1 in the W64A background at 10 and 14 DAP.
**Figure S9** Levels of sucrose (a), glucose (b) and fructose (c) in developing endosperms of WT, o2, oen1‐1, o2;oen1‐1 in the W64A background from 8 to 24 DAP.
**Figure S10** Alignment analysis of amino acid sequences of the three SUS proteins.
**Figure S11** Kernel phenotypes of WT and single, double and triple mutants of the three Sus genes.
**Figure S12** Levels of sucrose (a), glucose (b) and fructose (c) in 12DAP endosperms of WT and single, double and triple mutants of the three sus genes.
**Table S1** Primers used in this study.
**Appendix S1** The promoter information of Sh1, Sus1 and Sus2.

**Supplemental Dataset File** Original data and details for all statistical analyses in this study.
