# Supplementary material for: Transactivation of Sus1 and Sus2 by Opaque2 is an essential supplement to sucrose synthase‐mediated endosperm filling in maize
Source: Plant Biotechnol J. 2020 Mar 26;18(9):1897–907. doi: 10.1111/pbi.13349 (PMC7415785; doi:10.1111/pbi.13349)
Supplement: Supplementary file 1 — Figure S1 The K0326Y‐oen1 mutant generated by EMS‐induced mutagenesis of K0326Y. Figure S2 The plant phenotype of WT, o2, oen1‐1, o2;oen1‐1 in the W64A background. Figure S3 SDS‐PAGE analysis of zein (upper) and non‐zein (lower) proteins of mature kernels of WT, o2, oen1‐1, o2;oen1‐1 in the W64A backcross population. Figure S4 The phenotype of oen1‐1 in the W64A background. Figure S5 Genetic complementation test of oen1‐1 with sh1‐ref and oen1‐2 alleles. Figure S6 Kernel phenotypes of WT, o2, oen1‐2, o2;oen1‐2 in the W64A background. Figure S7 SDS‐PAGE analysis of zein (upper) and non‐zein (lower) proteins of mature seeds of WT, o2, oen1‐2, o2;oen1‐2 in the W64A background. Figure S8 SUS activity in developing endosperms of WT, o2, oen1‐1, o2;oen1‐1 in the W64A background at 10 and 14 DAP. Figure S9 Levels of sucrose (a), glucose (b) and fructose (c) in developing endosperms of WT, o2, oen1‐1, o2;oen1‐1 in the W64A background from 8 to 24 DAP. Figure S10 Alignment analysis of amino acid sequences of the three SUS proteins. Figure S11 Kernel phenotypes of WT and single, double and triple mutants of the three Sus genes. Figure S12 Levels of sucrose (a), glucose (b) and fructose (c) in 12DAP endosperms of WT and single, double and triple mutants of the three sus genes. Table S1 Primers used in this study. Appendix S1 The promoter information of Sh1, Sus1 and Sus2. [file PBI-18-1897-s003.docx]

**Supporting Figures and Legends**


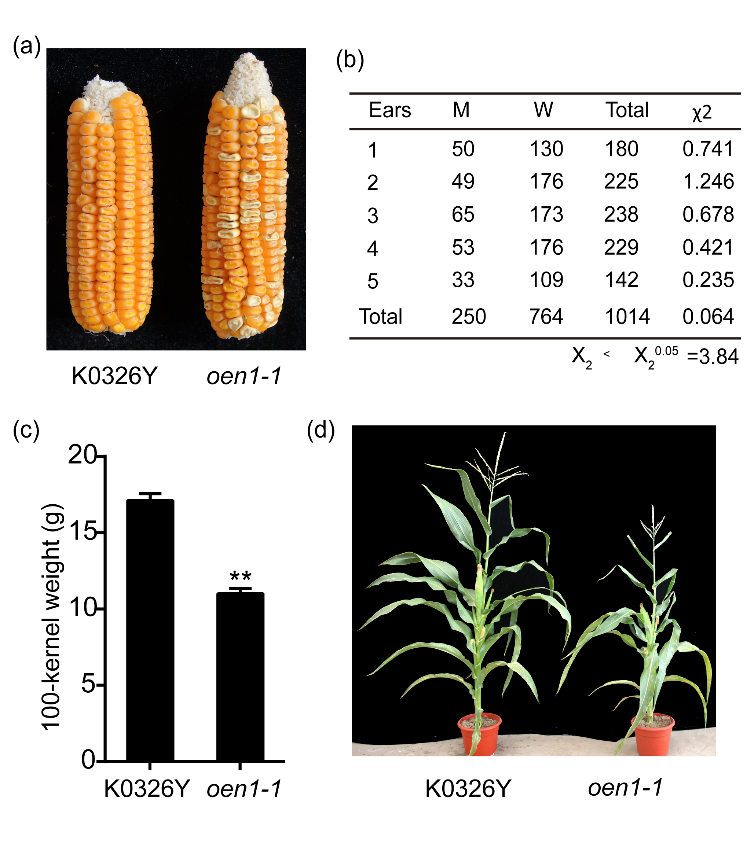


**Figure S1.** The K0326Y-*oen1* mutant generated by EMS-induced mutagenesis of K0326Y. (a) Phenotypes of the K0326Y and K0326Y-*oen1* segregating ears. (b) Chi-squared (χ2) test showing the single gene recessive inheritance of K0326Y-*oen1* in the segregating ears. The kernel number of WT (W) and K0326Y-*oen1* (M) in each segregating ear is listed in the table. (c) The 100-kernel weight of K0326Y and K0326Y*-oen1* seeds. The data represent the mean and standard deviation (SD) of independent quintuplicate measurements. g, gram. The double asterisks represent an extremely significant difference (Student’s *t*-test, P < 0.01) compared to WT. (d) Plant phenotypes of K0326Y and K0326Y*-oen1*.


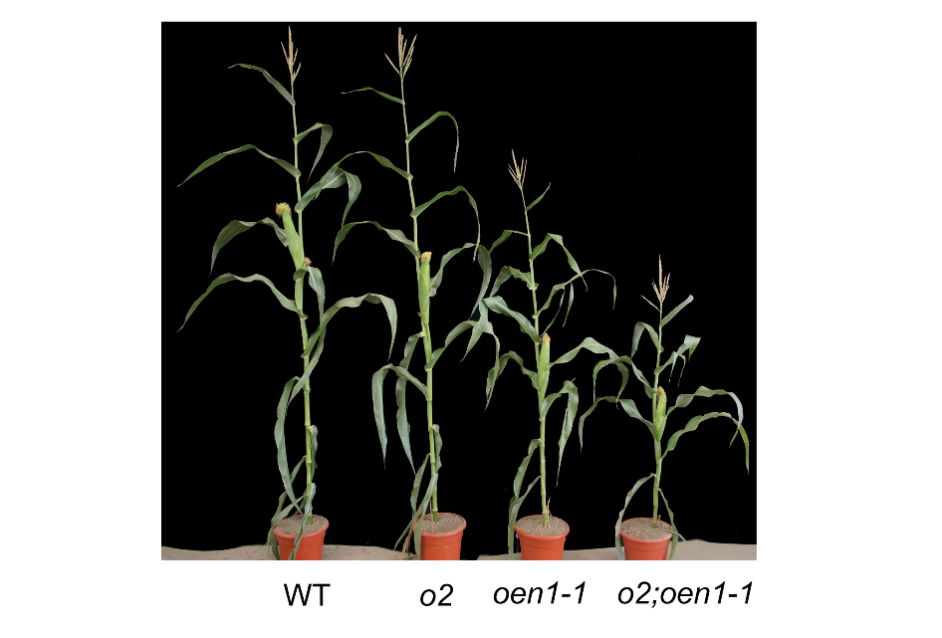


**Figure S2****.** The plant phenotype of WT, *o2*, *oen1-1*, *o2*;*oen1-1* in the W64A background.

**
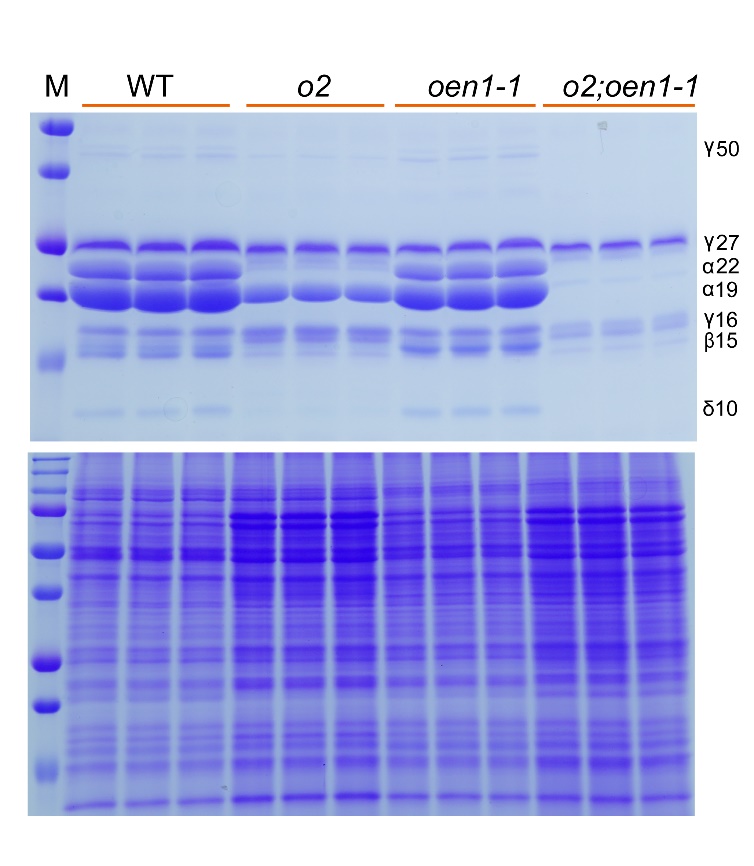
**

**Figure S3.** SDS-PAGE analysis of zein (upper) and nonzein (lower) proteins of mature kernels of WT, *o2*, *oen1-1*, *o2*;*oen1-1* in the W64A backcross population.


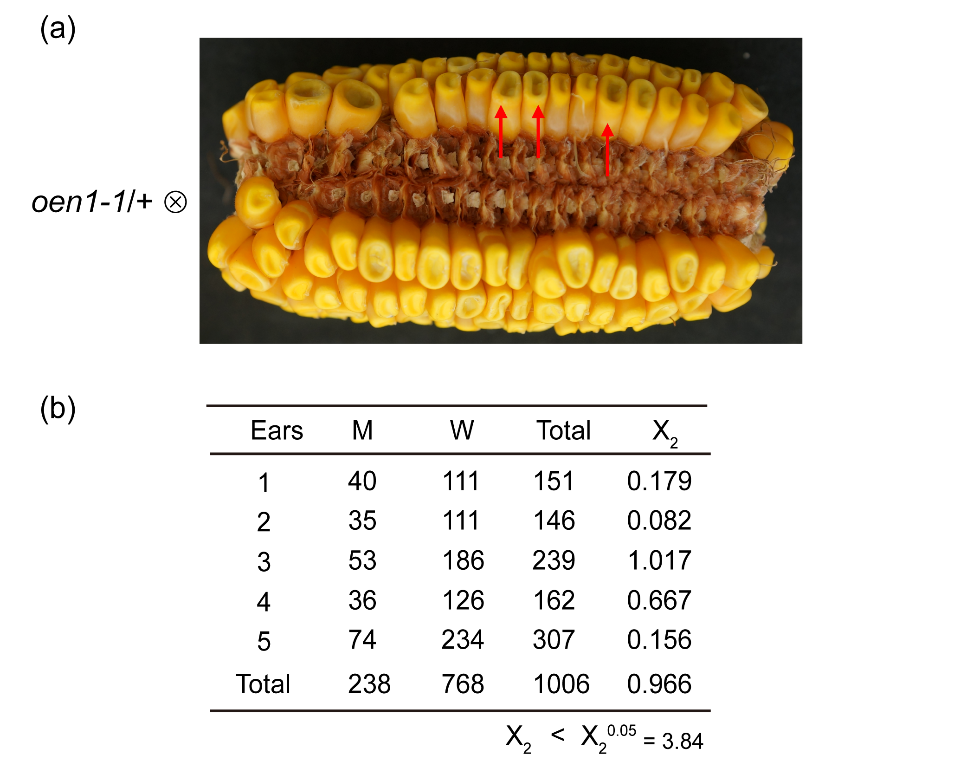


**Figure S4.** The phenotype of *oen1-1* in the W64A background. (a) An ear segregating *oen1-1* seeds in the W64A background. The red arrows show the mutant kernels. (b) Statistical analysis of the segregation of *oen1-1* seeds. The kernel numbers of WT (W) and *oen1-1* (M) in each segregating ear are listed in the table.


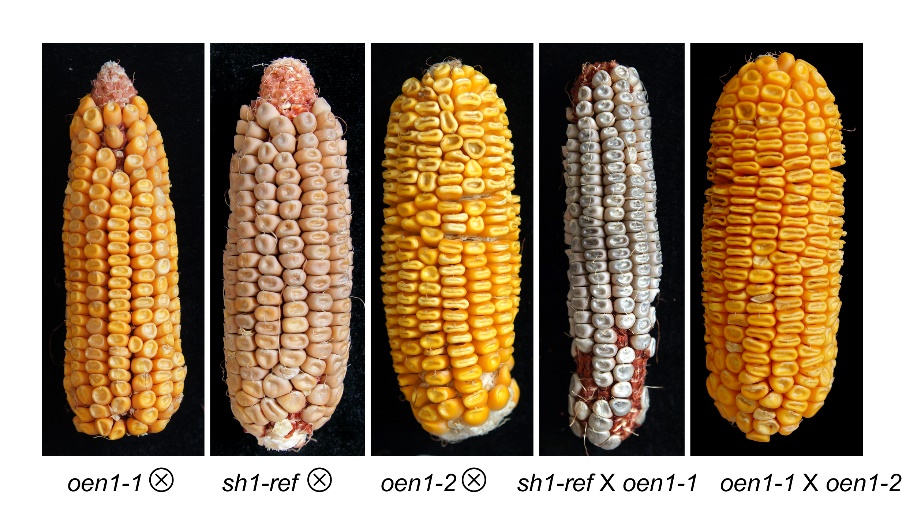


**Figure S5.** Genetic complementation test of *oen1-1* with *sh1-ref* and *oen1-2* alleles.


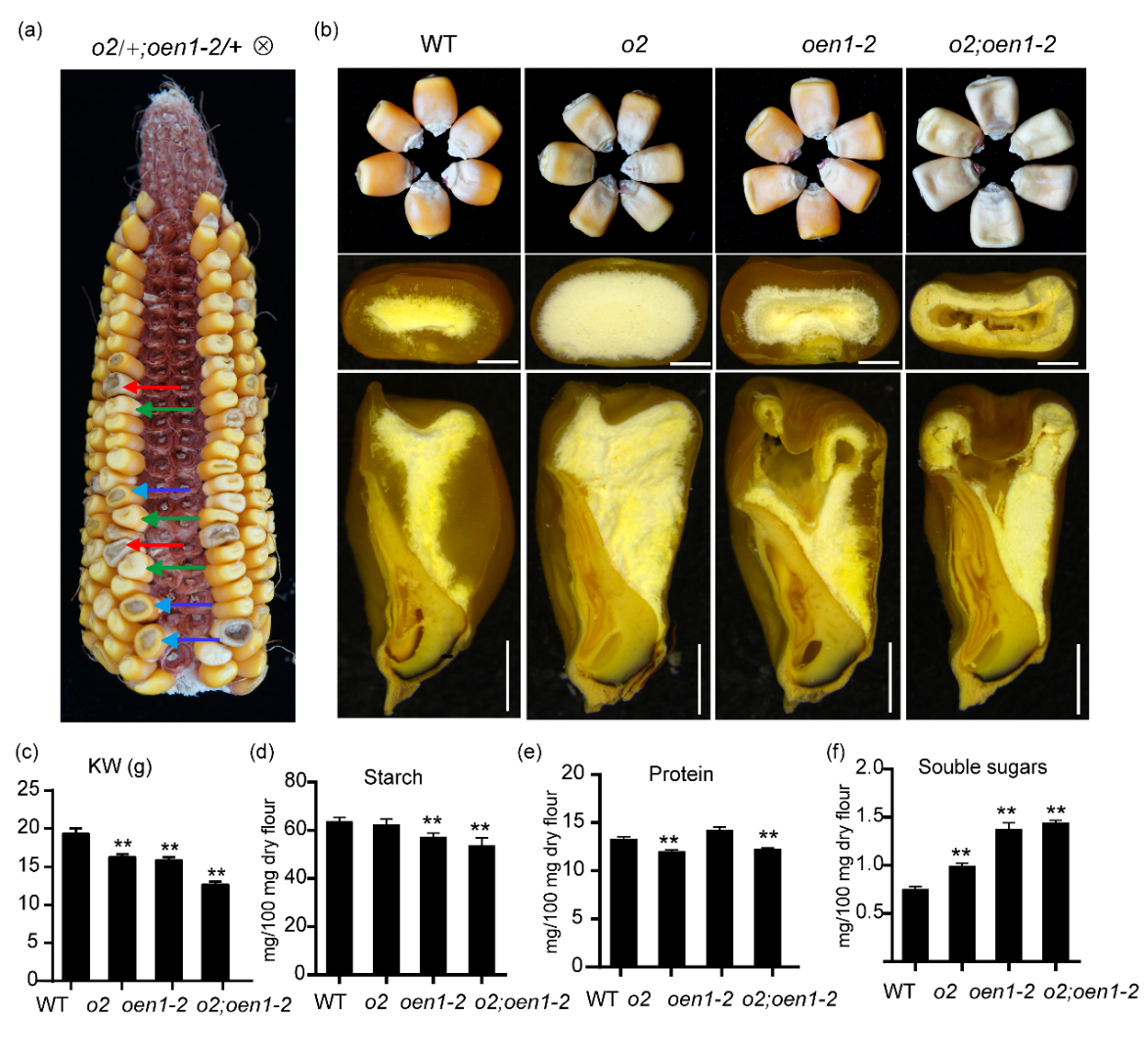


**Figure S6.** Kernel phenotypes of WT, *o2*, *oen1-2*, *o2*;*oen1-2* in the W64A background.

(a) The F_2_ segregating ear of W64A*o2* and W64A-*oen1-2*. The blue arrows show the *oen1-2* mutant kernels; the green arrows show the *o2* mutant kernels; and the red arrows show the *o2;oen1-2* mutant kernels. (b) Kernel phenotypes of WT, *o2*, *oen1-2*, *o2*;*oen1-2* in the W64A background. Top panel, kernel phenotypes; middle panel, transverse sections of kernels; bottom panel, longitudinal sections of kernels. (Scale bars = 2 mm). (c) The 100-kernel weight of WT, *o2*, *oen1-2*, *o2*;*oen1-2* seeds. (d) Starch content of WT, *o2*, *oen1-2* and *o2;oen1-2* in 100mg dry flour. (e) Protein content of WT, *o2*, *oen1-2* and *o2;oen1-2* in 100mg dry flour. (f) Levels of soluble sugars of WT, *o2*, *oen1-1* and *o2;oen1-1* in 100mg dry flour. The data represent the mean and standard deviation (SD) of independent triplicate (e) or quadruplicate (d and f) or quintuplicate (c) measurements. g, gram. The single and double asterisks represent significant difference (Student’s *t*-test, P < 0.05) and extremely significant difference (Student’s *t*-test, P < 0.01) compared to WT, respectively.


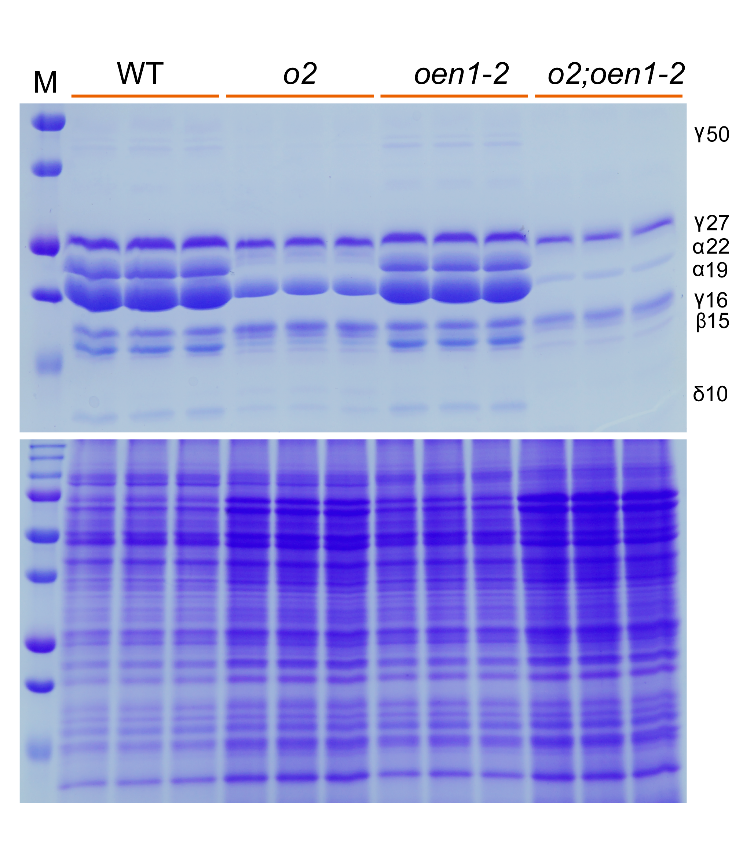


**Figure S7.** SDS-PAGE analysis of zein (upper) and non-zein (lower) proteins of mature seeds of WT, *o2*, *oen1-2*, *o2*;*oen1-2* in the W64A background.


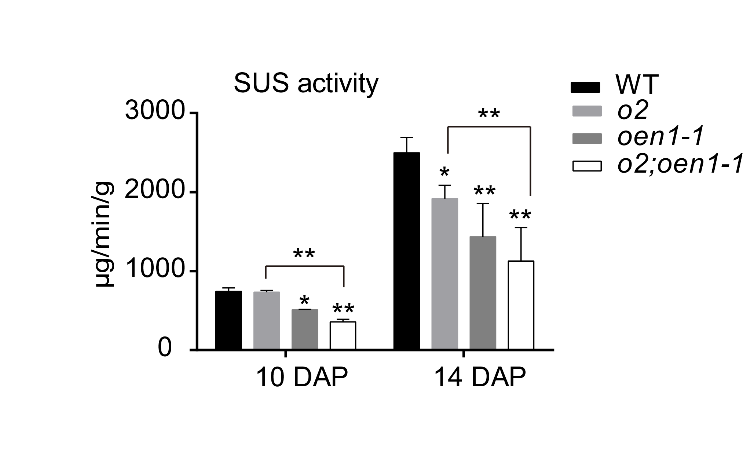


**Figure S8.** SUS activity in developing endosperms of WT, *o2*, *oen1-1*, *o2*;*oen1-1* in the W64A background at 10 and 14 DAP. The data at each time point represent the mean ± SD of six measurements. The production of 1 μg of sucrose in 1 min per gram of tissue is defined as one unit of SUS activity (μg/min/g). The single and double asterisks represent a significant difference (Student’s *t*-test, P < 0.05) and extremely significant difference (Student’s *t*-test, P < 0.01) compared to WT or *o2*, respectively.


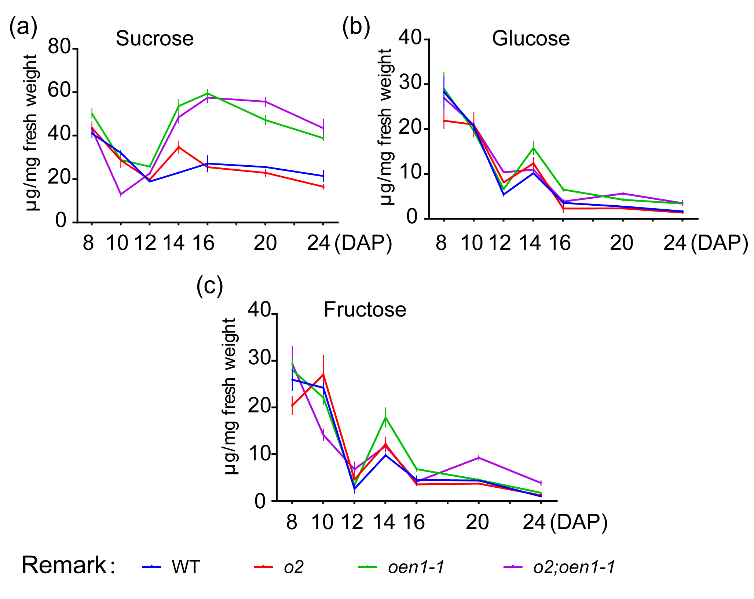


**Figure S9.** Levels of sucrose (a), glucose (b) and fructose (c) in developing endosperms of WT, *o2*, *oen1-1*, *o2*;*oen1-1* in the W64A background from 8 to 24 DAP. The data represent the mean and standard deviation (SD) of triplicate measurements.


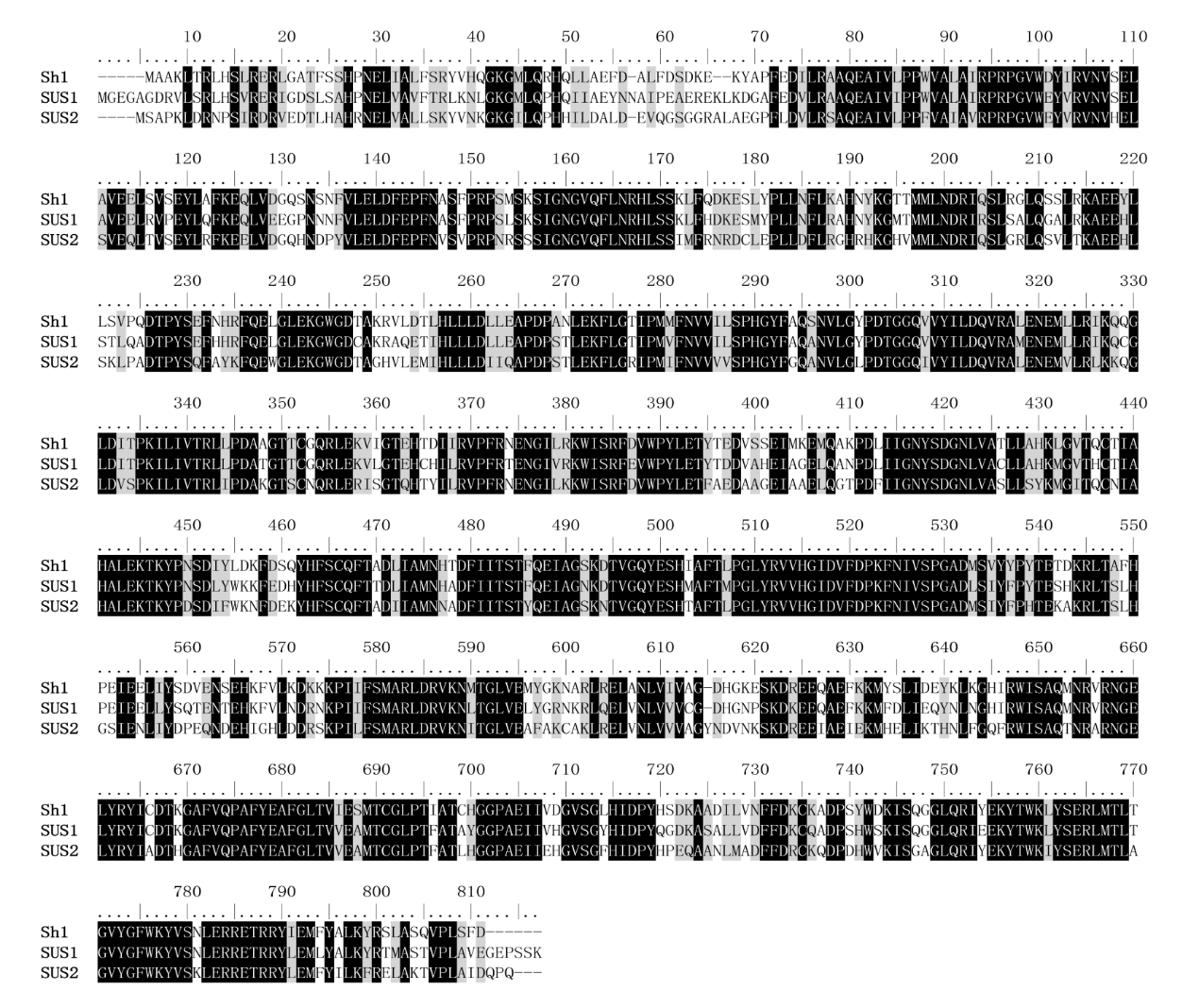


**Figure S10.** Alignment analysis of amino acid sequences of the three SUS proteins.


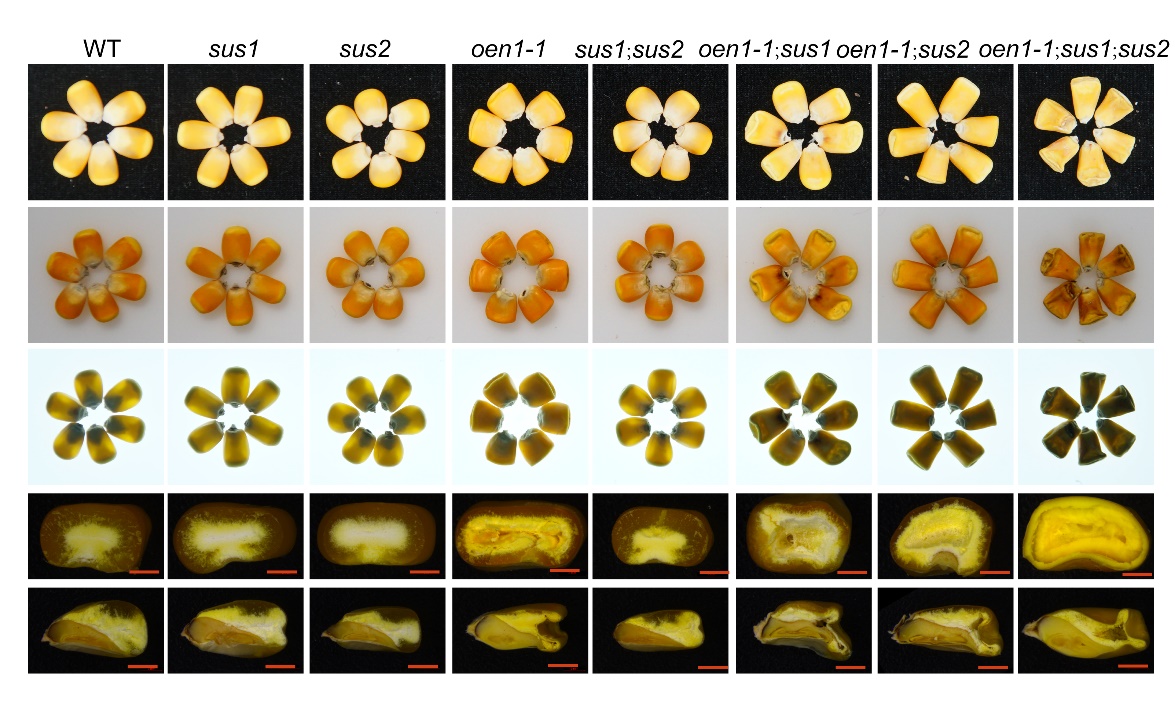


**Figure S11.** Kernel phenotypes of WT and single, double and triple mutants of the three *Sus* genes.


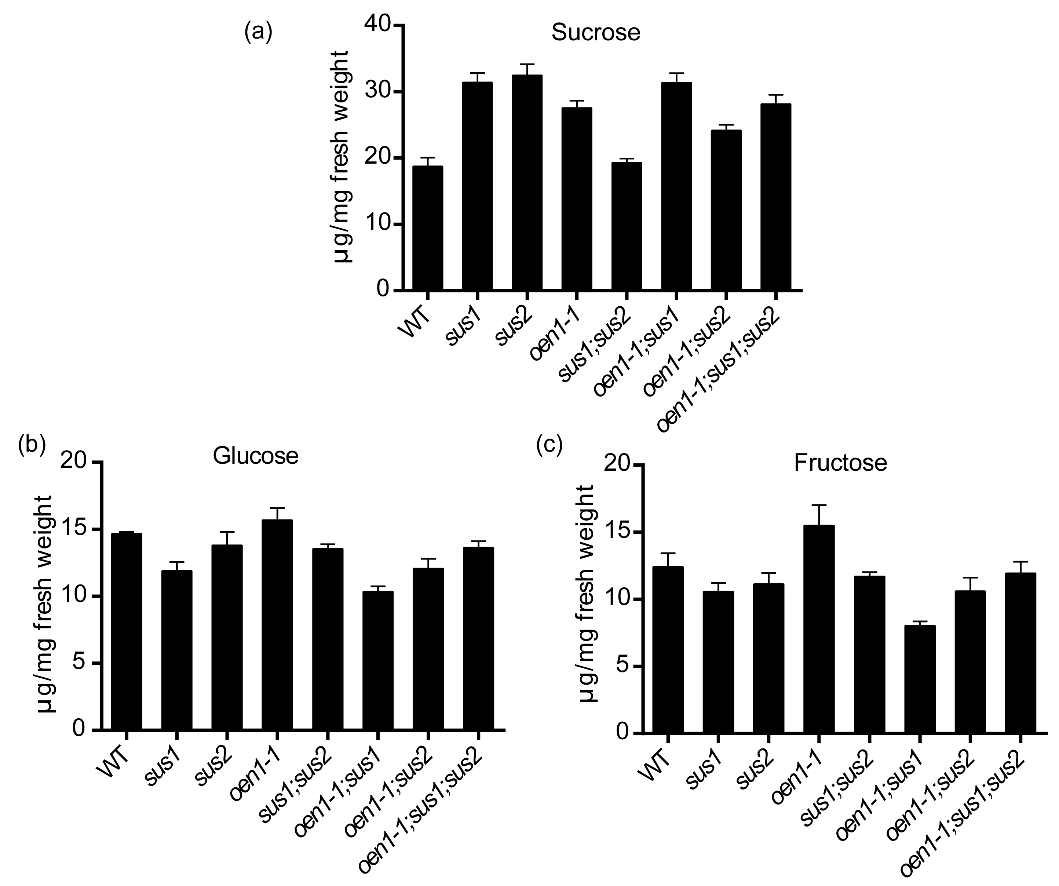


**Figure S12.** Levels of sucrose (a), glucose (b) and fructose (c) in 12DAP endosperms of WT and single, double and triple mutants of the three *sus* genes. The data represent the mean and standard deviation (SD) of quadruplicate measurements.

**Table S1. Primers used in this study.**

| Name | Sequence (from 5'to 3') | Useage |
| --- | --- | --- |
| Sh1qF | TGTTTCACCGCAATTCGCA | Quantitative  RT-PCR |
| Sh1qR | AGACAGGTGAACGAGCAGGC |  |
| Sus1qF | GGCGTTCGTTGCTGCTC |  |
| Sus1qR | ATTATTATTCAAGGAGCAACATCCC |  |
| Sus2qF | GTTGTGTGTTCGTTACTGTTTACTG |  |
| Sus2qR | GAGGGTTGAGGGCTGGTTG |  |
| UbiquitinqF | CTGGTGCCCTCTCTCCATATGG |  |
| UbiquitinqR | CAACACTGACACGACTCATGACA |  |
| W921 | TATGTTCACCAGGGCAAGGGAATG | RT-PCR |
| W924 | GCCAAGTACTCAGAAACACTCAGC |  |
| oen1-1F | AGCATAGTATAGCTGTTCTTCAGT | Identification of EMS mutation site in gDNA |
| oen1-1R | CCACAGCCAGCTCACTTACA |  |
| oen1-2F | CCTCCGCAAGTGGATCTCTC |  |
| oen1-2R | AAGCATCAGCCTACCACGTT |  |
| Sus1-MEMDF | AAGGATCTTCGGCGTGATGT |  |
| Sus1-MEMDR | GACCTGTGATGCATACCCGATA |  |
| Sus2-MEMDF | GCTTCGACACGTTCCTCGTA |  |
| Sus2-MEMDR | AAATGTATTCTTCATCCAAAAGCCT |  |
| 35SO2F | cccgggATGGAGCACGTCATCTCAATG | Effector  construction |
| 35SO2R | ggtaccCTAATACATGTCCATGTGTATGGCC |  |
| O2CDsF | catatgCCGCCGACGACCCATCAT | Protein  expression |
| O2CDsR | tctagaATACATGTCCATGTGTATGGCCCCA |  |
| PSh1F | cggtatcgataagcttCTGCAACGATACACGCATAACC | Reporter  construction |
| PSh1R | aattcgatctccaccgcggTGCAATTCGAGCTATACGGTCA |  |
| PSus1F | cggtatcgataagcttTTTGGGGCATGGATTGAGGT |  |
| PSus1R | aattcgatctccaccgcggCACAGCGAAACCGAAACGG |  |
| PSus2F | cggtatcgataagcttAGGATGGAGCATAGGGTGAG |  |
| PSus2R | aattcgatctccaccgcggTGGTCAGTGGGTCGTTTGTT |  |

**Appendix S1.** The promoter information of *Sh1*, *Sus1*, and *Sus2*.

***Sh1* promoter:**

-956

CTGTCAAACGCGACGGGATCGAGCTGCAACGATACACGCATAACCATGTACTATCGCCCCCCGGCTCCCCGCAAAGCGTTTGGGATCTCTTCATTTTTTTTTGTTTTTCCATGTCAGCGCCGCATAGGCAGCTCTGTTCTTGTTTGGAGCCAAGCCAGCCCCAGTCCACACCTCGCGGCACGCCCGATGCGAGTGCCGTTTGGCGCGCCTCATCATCGCTCACCGTTTGGGGGCCTGCCTCTGCCTTTCTGTTCTTCAAACGATGTCTCATGTCTGCGCTGGACAACTTTCTTGTTGCCGCCTGTCGCTTGCGCTGTGCTGACTGGACGCAGCTCCGGAGGTTTGGTTGTGCTTGGTTTTCGTAGAGAACTCGCCACTTGCCGCCCGCACGTTCTTGGTGTTTCCTCCTCCCCGCTGTGTTCTGCGCACGGGCTTTTTCTGAGAGACCCATGTTTCCCTTTTACTTTTATAAACAGTATACATGCTATGTTTCTAGAAGGAGGGGAAACCTAATCCCCCTAATCCAATGGCGGGGAGGAAATAGGGTGGGGTGGGGTGGGGGGAGGGAAATATCTCGCTACTTTTTAATCCGGACAAGCTCATTTGCGTTTGCGTCTGAATGATGATGACTGCAATGCTGATCGCACCTCGGGTGTCGGATCACCAGCTTTTGGCTGCTCTCACCAAATCAGCTGCAAGAAGATTAGAGCTCAAAAGAATTACAGAAAGAGAGCCTTTTTCTTTTCTTCCTTGTGGGGTTCCTTTCATTTCGTGCTCTCCTTTCTCTGCCAGCCAGTCCGTCCTTGCGTCCACTGCACCTGCACACAGGTCACCCCGACCCGCACTGTTCTAGACTCCATTAGAAAAAAAAAGGTCTGAACCTTTCCGAAACCAGCCAGCCATTGGTCTGGCAGGCCAGCATATGCTAATTGGATTTTTTTGCCGCATCATTGAGTGCGCCATCAGGATTTGGAAATC

+1

CTGGTTTTGAGTAATACAGTAATTTGGCATTATCCATTGCCGAATTCCCAAGCTCCGTCAGCTTGAACGTGGACCCCTACCATCTGCACCAGCTCGGCACCTCACGCTCGCAGCGCTAGGAGCCTAGGAGCAGCTGCCCGTCTATTTATTGGTCCCTCTCCCGTCCCAGAGAAACCCTCCCTCCCTCCTCCATTGGACTGCTTGCTCCCTGTTGACCATTGGGGTATGCTTGCTGCCTTGCTCTCCTGTTCATCTCCGTGCTAAACCTCTGTCCTCTGGGTGGGTTTTTGCTGGGATTTTGAGCTAATCTGCTGGTCCCGGTAGAAAAGATCATGTCCCCTGACGAGCTCAAGCGCTCGCCTTAGCCGCGTCCTTGCCCCCCGCCATTTTTTGCGGTTTCGGTGTGTTCCCGTGACTCGCCGGGTGCGTCATCGCCTGAATCTTGTCTGGGCTCTGCTGACATGTTCTTGGCTAGTTGGGTTTATAGATTCCTCTGATCTAAACCGTGCCTGTGCTGCGCACAGAACTCTCCCCTGTCCTTTCCTGGGGTTTTGGTTACGTGGTGGTAGTAAGCTTGGATTTGCACATGGATAAAGTTGTTCTAAGCTCCGTGGGTTGCTTGAGATCTTGCTGTTATTGCGTGCCGTGCTCACTTTTTTTGCAATCCGAGGAATGAATTTGTCGTTTACTCGTTTTGGTGGATTATTAGCGCGAAAAAAAAACTCTTTTTTTTTTGTTCTTTTACTACGAAAAGCATCTTCTTGGATTTTGCTATCTTCTTTTACTACGAAAAACTCTTGAGTCTAGGAATTTGAATTTGTGATGTCCATTCTTGCAGTGCGCTGTGCTTTATTGGGAAGCCAAATCCTATTATTTTCTGCCTCTAGGGTCTGAATGGAATCAGTACTCTTGAGACAGAAAATCAATCCAATCAAGTTGATTTCTTTCTTTAAAAATATTATCACAGAACTAAGTGCTTGTGCGGAATCAGTACTGGCTTTTGTTTGGTGGAGGATCAATACTTGCTTTTGTTTGGGGGTGGCAACTGTTTTGCTATAAGATTCCATGTGTTCCTGTTGAGATGAATCATATATAGTATAGCTGCATACTACAAATCTGTTTTTCAAATTTAGGTTGCTTTGGCATGATCTATTTTTTTGTCAGACAGACTTTCTAAGTGGTAGCTCTTGATTTCTTGTTCTTGTACAACTGGTGCTGCTGAATCTTGACCGTATAGCTCGAATTGCAGTATTCTGAACCATCGAGCCATG

ATG start codon GTATAGCTCGAAT 5`UTR **ACGT** O2 box core sequence

Probe in *Sh1* promoter: GAATTCCCAAGCTCCGTCAGCTTGAACGTGGACCCCTACCATCTGCACCAGCTCGGCA

Mutant probe in *Sh1* promoter: GAATTCCCAAGCTCCGTCAGCTTGAAATTGGACCCCTACCATCTGCACCAGCTCGGCA

***Sus1* promoter:**

-1100

GGGGCATGGATTGAGGTATGGATTTAATTCCAATCCATGCTAATCCAGAGCGGGATTGGTGTAAACGAACAAAGCCTAAAGGATCCGGGTTGGATCCCGAGTAGATCCACCAGCAAAAGTGGGGGCGCCCGCCCCTTTGCTTCCAACCCACGCAAAAACCCGGATCCGATGAATGACCCTCCACCTCCACTTCCTCCTCACGGGCGGTCGCCTCGTCGTACAGACGAACAGAAACAAATGGCAAGCAAAAAAATATACAATACCAACAAAATACCGCGAAAGAATGCTACGGTCACCGGGGTGCATCTGCCGCCAATTCGACAGCCGTGTGTCTCTGTCTCTTCAAAGCACGGAAAGGCAGGCCGCCGGGGCGAGTGTGGTTTTTTTCCTTGTGTGCAAGCATTAGGGTGCAGGTACCAAAACTGCTGCGCGTCAGAAACCATGGGGTCATCAGCTTTGGCGGATTTTGGAGGCGGTGATGTGCGCTCCCTCTGTTTTCTCATGTACCAAGGTCAGAAGGTGACGCGGCGTCGGACCGTGTGCATTTATTTATTTATTATTACAACAGCGAGATTACCGTCCGAATGCCAAATTTATTACTACCGAAACAAAAAAAAATACCAGCCTCGTCTGTTCTACTTCTAGT**ACGTACGTACGTACGT**ACAGGGCGCGTCAGCACTGCTTGTGCTTGAGGACAAAAAAGGAGTGGTTTTTAGTGGCAGAAAGTTCCCGCTACTTGTCACGCATTAAACCTCCGCTGATTTAGGAGTAGAAACCTTCTTTCAAAAAAAAATCTTTGAAGATAAATAAAAAGAGAGACCCAAAATACTCCATCCCAAGCTGATCATATCCGATCATACCTGGACATGTAAACAAAAAATCCATCCGTAAACCAGCATCAGATGAAGAAAGCAAACCCAATGCACTACCAAAAGGAAAGAAAGAAAAAATTCCTAATCTTTCTTATCCCTCACCTACCATTTTTTTAAAAAAATCTAATACTACATGGGCAAGGGACGAAGGTGGTGGGAGCAAAGAGGCCCCCACCCCTGCCCCCGCAATGCGTGTATAAAACGGGGCCCGGGGCTGCCGCTCAACTCACCCATCCATTCCACCTCCGTT

+1

CACCCCGTCCATTTGATTTGCGTTCACTGCGTTGCGTTTCCTTGGAGGGGATTGTTCTCTCCTCTCCTTTGGATTGGAGGTCCCTCCTTCTTCTCCTCTCTCTCTCAGAGGAAGGTAATGCAATGGGGGTGCTCTAGCCCTCTCCTCCTCTGCTCCTGCCGTCCTGCGTGGGTGCGTGCGTGGACGCTGGTCTGCTGTCCTGCTCCGGGCGAAATGCCGCCGCCGCTCGCCGTGACTGATGCTCCTCCTCCTGGTCGTGTCCGAACGCGCCGGACGGTGCCGCCGGTAGCATGTAGGAACTATACGCTCCGCTCGGACCTGGTCATGATCGATCGATGGTGTAGGGGAGGTCATGCCGAATTTTTTCGCCCGGTTCTCTGAACCTGCCGCCACTCTGCAGGCGGGGGAGGTGCTGCCTCTGCATGCAGTTGCTTTCCTTGCAGCTATAGCCAAAACAAACAAAACAAAAGACGGGTTTTTTTACGGCTGTTGTACCAGCCTACCAGGGGATAGCTAGCGACTAGCGTCCGTGAGATCCAGAGAACTATTGTCTGCCACTTGACGAGATCCTAGGGGAGCGGAGGGAGCCTGCTTATCCGCGCAGGAACGACGGTGTTAGCTTCTGGATCTGGGGTTTACTGAGCACCAAGGCTCTTGCAAACTCCGGGTTTAAGGTTTCATATATATATATTTTTTGCATACTCCCGAGTTGGTACCAGAACGCTAGTAGATCCGGCGCTTCTTTGTTTCCTCTTTAATTAACCAATATTTTTGCAAGAAATATTTCTGTGACGATTTTCCTCGGATAAGTTGTTGCCTTGTTGGCTTTATCCGTCCGGATGCCCCGCCATGTGCTCCTTGTCTCGGGACTCGGAGACAGGCTGACAGTCCTGGGTTCTTGTGCTAATGGCCATGGTTAACTTACGTTAATCCTGCGCCTTATTATCATCATCTAGGTGTAATTACTTCCGTTTGTGCTAATCACCTCGTGATTGCCTGCAATAAGGAACCCCAGCCCCCCCACCTTGCTGATGCCGTGCTTTTCTAGAGAAGTACCCTGCCGCGTCACGCTTCGTCCTCGCCTGCTTTGGATTCAACGGCTTTTTGGAGGCCGAGGCCATTGGTGCCATGCGGCCAGCCCTTTTCTTCTCCATGGTTCCCATCGATGTGTTTTTGTTCGGTTCTCTCGTCAGATCTGTATAAATAGGCGCCTCCCTTCTCCGCCATTCCTCGGTCCTCTGAAGCGTTTCAGTTCATCGATTCAGTTCTTGTATGCCTCTTGTTGTATTGGTTGTTTCTTCTTTCTGGTCTAGGTACTAGGACTATAGTACCAGGATCTGAGTCGTTTTTTTTTGGGTCTTGCTCCTGTCTGCCGTTTCTTTCCCCCCTTCCAGAGTTAGGTTCTGTTGGTTTCTTGCCTGCAATATAGTTTCGTGGCGCAGCGTCAAGGGTGTGTCGAGACTTAAAAGACTGGTTGTTGGCAGTTGGGTTTATGTCTTGCTGGAGGGGTTTTAGTTTTAGGATGTCATCGCAGCTTTTATGTCAACGGATCTGAACCGTTTTTCGGTTTCAGTTTTCTCTGCTGACTGAAGCTTCCGCTTCCGTTTCGGTTTCGCTGTGCAGGCCTGAGGATCCAGGAAGAGGACAGCAATG

ATG start codon GTATAGCTCGAAT 5`UTR **ACGT** O2 box core sequence

Probe in *Sus1* promoter: GGGTTCTTGTGCTAATGGCCATGGTTAACTTACGTTAATCCTGCGCCTTATTATCATC

Mutant probe in *Sus1* promoter: GGGTTCTTGTGCTAATGGCCATGGTTAACTTAATTTAATCCTGCGCCTTATTATCATC

***Sus2* promoter:**

-1207

AGGATGGAGCATAGGGTGAGGAGCCGACTAAAAAAATTTCTATGCTAATCCTTATAAAAAAGTACAACAGAGAAATGTTTACTCCTAACTTTTAGTGTAAGTGTAGAAACGGTTCTCTTCTACTCATACACGAATCCATTGACATTTTAGGCCTTCTTTAGAACACAAGAAGGAATCAACAGGGAATTTTTTTCGTGTTTCAAAGGAGACCTTAACCGTTCGACTGATGGGAGTGATTCTCTTGGAAAGAGTTATTAACATACTTGTGCATGCTATTTTGTTCCAAAAAAAAAAAACGATTGCAAATACATTTGTTAACAAGTTCGTTTAAATATATAAACAAAAGCATTAATATTTTTACACTACATAAGCAAATTATAAAATATAATTTAAAACAAATTAAATAATGTTTTTTAAATGTTATAATTGTTTACGTTTTTATTTATAATTAATTTAATTTGAGATACTTTGATTTGTGCTGGCATTGTTCTAATGAGACTACGACAGCATTGTGTGTCCTGTGTCGTTGCCACTGCCACACTGGCCTACTATTCTATAGCCTCTGTCC**ACGT**CCCCGGCCACGTCTCTGTCGTTTTCTCTCCAGAAGCGTCTCGTCGCCGCTCCCTCGCTACACCCCCGACCCCTCACAGAAACAAACAAGCCCAGTCCAGGGCAAAAACTAAACAAGAGGGGTGGTGAAGAAAAGGGAAACGGAACCATCTCGTGGCAGAGCGCCGATCTGGACGGCGGAAGCAAAAGCAAATGTGATGGTTATCCGCACTAATGGGAGCCACGTTAGAGCGGGGTCCATTGGTCGGTCTCGACCAGTAGTGCCCGTGCCCTCCACCTGCTGCGCCTTTTCTTCCATCCCCACGCGAACGTGGGCATTCTTGGCATGCCGGTGCTGCAAGATACC**ACGT**ATCTGGACCCAGAAGTCAGCGAGGCACACCCTAGCGTTCCTTCTAGACGTCACGCCACGCCAGGGAACGAAACGACTCCTTGGCTAGATCCGCTGACTGCCTCTGCCGGAACCCACGCACCCGCGTGCTACGCGCGGGGTCACACAAACACAACAGCGTCACACGCACACACGTACGCGATGGTCGCCCCCACCCCGCCTGCTACGCGTCCACAGACAGGGCGCCACGGCGAGCGGTTGCACGCCGCGTGCCTTGTATAAAGTCGGGGGCCGCCTTCCGCTGTGTTCTCGCATC

+1

TCTCGCCACTTCGCAATTCGCCAGTCGCCACCGGCACCACCACACCCACCCTCGCGGCCGCGAACAAACGACCCACTGACCACTGGCGGGCTGCACGGCAGCCATG

ATG start codon GTATAGCTCGAAT 5`UTR **ACGT** O2 box core sequence

Probe 1 in *Sus2* promoter: TCTATAGCCTCTGTCCACGTCCCCGGCCACGTCTCTGTCGTTTTCTCTCCAGAAGCGTCT

Mutant probe 1 in *Sus2* promoter: TCTATAGCCTCTGTCCCCCCGGCCCTCTGTCGTTTTCTCTCCAGAAGCGTCT

Probe 2 in *Sus2* promoter: TCTTGGCATGCCGGTGCTGCAAGATACCACGTATCTGGACCCAGAAGTCAGC

Mutant probe 2 in *Sus2* promoter: TCTTGGCATGCCGGTGCTGCAAGATACCAATTATCTGGACCCAGAAGTCAGC
