# Supplementary material for: Transactivation of Sus1 and Sus2 by Opaque2 is an essential supplement to sucrose synthase‐mediated endosperm filling in maize
Source: Plant Biotechnol J. 2020 Mar 26;18(9):1897–907. doi: 10.1111/pbi.13349 (PMC7415785; doi:10.1111/pbi.13349)
Supplement: Supplementary file 3 — Supplemental Dataset File Original data and details for all statistical analyses in this study. [file PBI-18-1897-s004.docx]

**Supplemental Dataset File contains Supplemental Dataset 1, 2, 3, 4, 5, 6 and 7.**

Supplemental Dataset 1: Data related to Figures 1b, S1c and S6c.

Supplemental Dataset 2: Data related to Figures 1e and S6f.

Supplemental Dataset 3: Data related to Figures 1c, 5c and S6d.

Supplemental Dataset 4: Data related to Figures 1d and S6e.

Supplemental Dataset 5: Data related to Figure 3c.

Supplemental Dataset 6: Data related to Figure 4a.

Supplemental Dataset 7: Data related to Figures 3a, 5b and S8.
